# Comparative Dynamics Enables Discovery of Embedded Bacterial Ferredoxin Domains in Large Redox Enzymes

**DOI:** 10.1002/prot.70004

**Published:** 2025-06-19

**Authors:** Jan A. Siess, Vikas Nanda

**Affiliations:** ^1^ Department of Biochemistry and Molecular Biology Robert Wood Johnson Medical School and the Center for Advanced Biotechnology and Medicine, Rutgers, The State University of New Jersey Piscataway New Jersey USA

**Keywords:** anisotropic network model (ANM), comparative dynamics, ferredoxin domains, protein evolution, protein structure dynamics

## Abstract

Bacterial ferredoxins are small iron–sulfur binding proteins that function as soluble electron shuttles between redox enzymes in the cell. Their simple 2×(β–α–β) fold, central metabolic function, and ubiquity across all kingdoms of life have led to the proposal that ferredoxins were likely among the earliest proteins. Today, ferredoxin‐like folds are embedded in large, multidomain enzymes, suggesting ancient gene duplication and fusion events. In some cases, these embedded domains may have scant sequence or even structural homology to soluble counterparts, challenging the use of traditional phylogenetic tools to establish evolutionary relationships. In this study, we identify fragments of bacterial ferredoxins within larger oxidoreductases by integrating comparative sequence, structure, and dynamical attributes. Dynamics are computed using an elastic network model and analyzed for similarity of major normal modes. Using comparative dynamics, fragments of ferredoxin domains are found within larger proteins, even in cases of limited structural homology. This study also reveals a non‐linear relationship between dynamical and structural similarities, suggesting that protein dynamics are more constrained than structure through evolutionary time. We propose that dynamical similarity is indicative of functional similarity, and since nature selects for function, that the inclusion of dynamical similarity, in addition to sequence and structure similarities, provides a more robust framework for inferring homology. Inclusion of dynamical attributes in comparative analysis will lead to a greater understanding of the deep‐time evolution of modern protein nanomachines.

## 1 Introduction

Modern oxidoreductases (EC1 enzymes) are large nanomachines essential to driving electron‐transfer reactions. They contain prosthetic groups comprising a select set of transition metals that facilitate catalysis and are present within all forms of life [1]. Despite their ubiquity, the deep‐time evolutionary origins of these enzymes remain poorly understood. It is hypothesized that modern oxidoreductases represent an assembly of smaller folds that had undergone a series of gene duplication, combination, and sequence divergence events over billions of years [2, 3, 4]. Uncovering the trace of these ancient processes is difficult due to the statistical limitations of current phylogenetic methods over such immense timescales.

Traditional sequence‐based phylogenetic methods, applied at both the gene and genome levels, have provided deep insights into redox protein evolution [5, 6, 7, 8, 9]. Yet, these methods often struggle to resolve ancient divergence events spanning billions of years [10, 11, 12]. Comparative three‐dimensional structure analysis [13, 14, 15, 16], especially when combined with functional insights from cofactor interactions [4, 17, 18, 19, 20], has also been employed to tackle this challenge. However, in large multidomain oxidoreductases, where domain fusion and insertion events obscure homology, purely sequence‐ or structure‐based approaches fail to identify distantly related domains. As a result, there is a clear need for complementary methods that can elucidate these hidden evolutionary relationships.

Recent structure‐cofactor interaction network analyses have proposed that close spatial proximity of distinct folds may represent “fossil evidence” of divergent evolution in large proteins [17, 18], arising from domain duplication and fusion followed by extensive diversification. One example is the proposed transition between the bacterial ferredoxin fold and the Rossmann‐like family domains [17]. Yet, these studies often rely on strict structural proximity cutoffs (e.g., microenvironments within a 15 Å sphere centered around the cofactor), which may overlook domain boundaries, discontinuous segments, and domain insertions. While cofactor‐driven inference has been indispensable toward inferring deep‐time protein fold evolution, a different approach is needed—one that can detect evolutionary signals even when structure and sequence similarities have been heavily eroded.

Although protein domains are often described in terms of independent folding, modular function, or evolutionary selection [21, 22], no single definition can fully encapsulate a protein domain. Traditional domain identification methods—whether homology‐based [23], ab initio [24], or structure‐focused [25]—have become more powerful with the rise of large sequence databases and both machine‐ and deep‐learning‐driven structure prediction tools [26, 27, 28, 29]. Still, discontinuous or highly modified domains remain difficult to characterize. Notable, recent, approaches that incorporate protein dynamics‐defining domains as regions undergoing collective movements‐offers a new way to investigate otherwise cryptic domain boundaries [30, 31].

Protein structure tends to evolve more slowly than protein sequence [32, 33, 34], but the functional motions of a protein—its dynamics—could be even more conserved if those motions are pivotal for its activity [35, 36]. We propose that low‐frequency, large‐amplitude normal modes play a particularly important role: if these global motions underpin function, then they may remain conserved over immense evolutionary timescales, even when sequence and static structure have diverged [37]. If this is the case, dynamics may not only delineate domain boundaries of a large multi‐domain protein, but could enable us to detect homology among remote or cryptic domains beyond the structure and sequence level.

Comparative dynamics can be pursued using strategies at differing levels of granularity, from coarse‐grained elastic network models to all‐atom molecular dynamics (MD). Anisotropic network models (ANM) reduce a protein into a mass‐spring network where each residue is represented by the position of the backbone alpha‐carbon [38]. ANM computations are rapid, but do not explicitly consider chemical properties of the amino acids. In contrast, MD simulates explicit atomistic motion, capturing side‐chain interactions and solvent effects over time. Movement is calculated through the application of an empirical forcefield on all atoms, which incorporates bonded terms like bond stretching, angle bending, torsions, as well as non‐bonded interactions. Newton's equations of motion are then integrated in discrete time steps to generate trajectories depicting detailed structural dynamics at atomic resolution [39]. However, this level of precision comes at substantial computational cost, especially for large systems or long simulation periods, where most meaningful biological processes take place [40, 41, 42, 43]. ANM, conversely, while unable to replicate the same level of detail and resolution as captured by MD, has been shown to effectively predict functionally relevant, large‐scale motions at significantly lower computational expense [44].

Normal mode analysis (NMA) [45, 46, 47] is a mathematical approach that extracts collective motions, identifying essential modes corresponding to large‐amplitude, low frequency global movements in proteins. NMA can be applied to atomistic trajectories generated by MD simulations, or through simplified coarse‐grained models such as ANM. Despite differences in computational complexity and granularity resolution‐wise between these methods, both ANM‐based and MD‐based NMA identify similar essential modes [38, 48, 49]. By focusing on these large‐scale modes, which are known to mediate key functional transitions [50, 51, 52], we can discern evolutionary signals that might otherwise be masked.

In this study, we use ANM for its computational efficiency in analyzing very large, multidomain oxidoreductases. It also provides the requisite data for conducting comparative dynamics analysis, leading to the quantification of dynamical similarity between two structures. When the term “dynamical similarity” is used, we are referring to the extent to which two proteins (or protein subdomains) exhibit comparable large‐scale motions, as determined by overlap in their lowest‐frequency normal modes. Formally, we quantify this by comparing these modes—often through dot products or cosine similarities—to see how closely the direction and spatial displacements of the essential functional motions align between different structures. Because these large‐amplitude movements directly underpin function, dynamical similarity can highlight deep evolutionary relationships that may be missed by purely sequence‐ or structure‐based comparisons.

NMA‐based approaches have been illustrated in globins, where the first few normal modes are highly conserved across the family, reflecting shared functional motions [53, 54]. Because these large‐scale, global motions often coincide with essential functional rearrangements [37, 46, 55], and are robust against random mutations [56], it would necessarily follow that evolution should be selecting for conservation of the first few normal modes. Higher‐frequency modes, by contrast, tend to differentiate subfamilies, highlighting how dynamics both unites and diversifies proteins at different evolutionary scales [57].

Bacterial ferredoxins, in particular, provide an ideal test case for this framework. These small (typically 50–80 amino acids) iron–sulfur binding proteins are among life's earliest domains [58, 59], likely serving as electron shuttles in primitive metabolic networks. Their ubiquitous 2×(β–α–β) fold is found both as an isolated domain and embedded within large multidomain oxidoreductases, where repeated gene fusion and sequence diversification events may obscure direct sequence or structural homology, making their isolation and characterization challenging.

**FIGURE 1:**
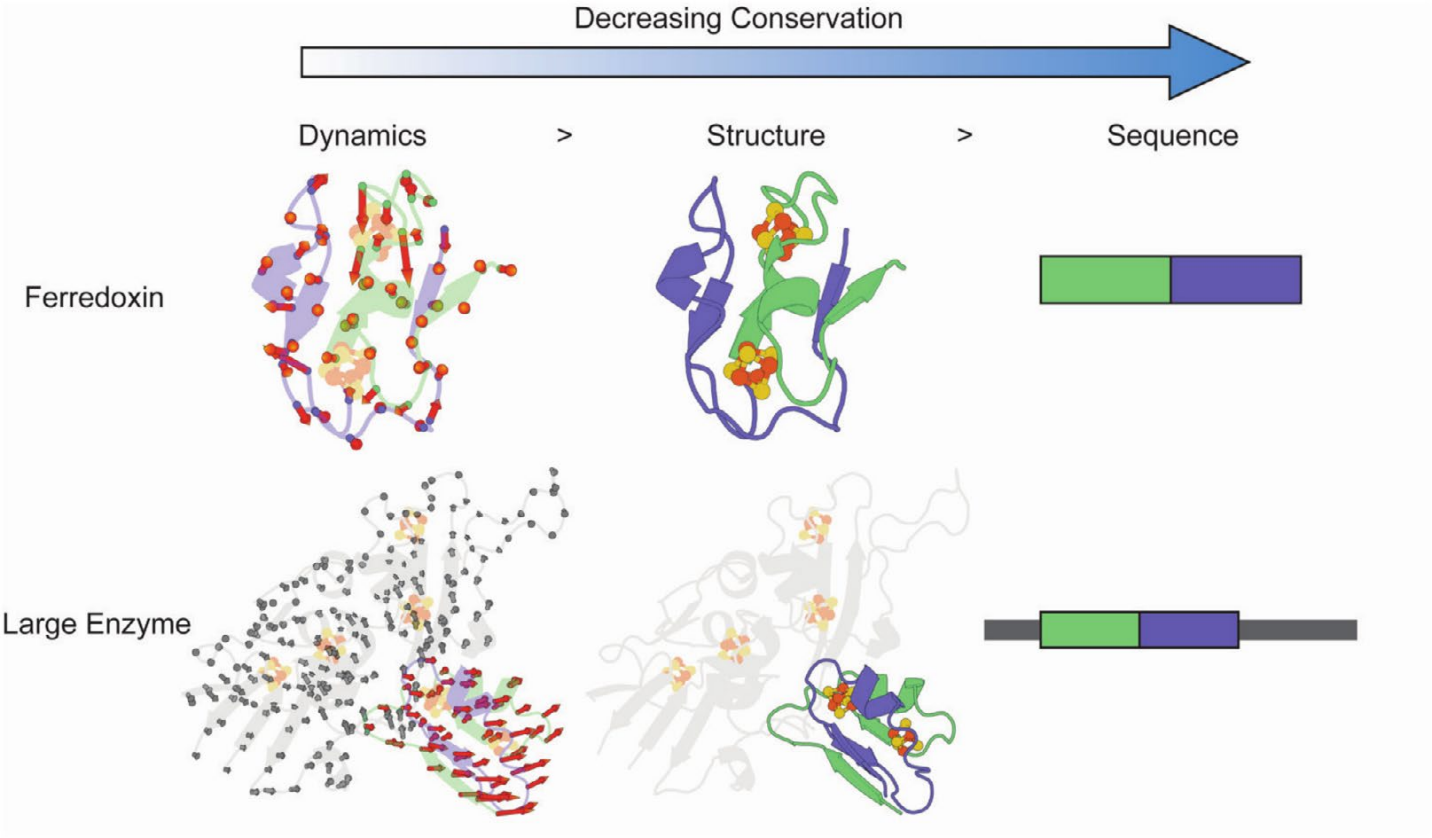
Ferredoxins embedded within larger structures exhibit similar motions to smaller, single domain ferredoxins: a structural analysis of a bacterial ferredoxin from *Clostridium acidurici* (PDB: 2FDN, top row) and a polyferredoxin segment from *Methanothermobacter wolfeii* (PDB: 5T5I, bottom row), illustrating homology through dynamics. Normal mode vectors obtained from an anisotropic network model (ANM) are used to generate per‐residue displacement vectors, which are shown to highlight the dynamic behavior of each residue. Dynamics of ancient domains are conserved in the context of larger protein environments, showcasing the similarity of motion between smaller ferredoxins and the larger polyferredoxin.

In this study, we integrate sequence, structure, and comparative dynamics to detect ferredoxin‐like domains buried in massive oxidoreductases. We focus on a small soluble 2[4Fe‐4S] ferredoxin from *G. acidurici* [60] and compare its essential modes with those of several larger oxidoreductases that contain multiple 4Fe‐4S clusters forming an electron‐transfer relay. Similar to approaches that identify repeats or subdomains based on local similarity in sequence and structure [61, 62], we incorporate an ANM‐based comparison of the lowest‐frequency normal modes. Notably, we find that dynamical overlap can reveal hidden ferredoxin domains—even when structural similarity is minimal—implying that the global, low‐frequency motions may remain evolutionarily constrained (Figure 1). By jointly analyzing sequence, structure, and dynamics, we offer a robust framework for investigating the deep‐time evolution of protein domains, shedding light on how ancient building blocks persist within modern protein nanomachines [18].

## 2 Methods

### 2.1 Anisotropic Network Models (ANM)

Coarse‐grained NMA was performed using an ANM in the ProDy package (Version 2.3.1) [63]. The analysis included the main‐chain carbon‐alpha (Cα) atoms of a query ferredoxin structure and three large oxidoreductase targets, each containing putative ferredoxin‐like domains. Within the ANM framework, each Cα atom is represented as a node in a uniform mass‐spring network, where springs of constant *γ =* 1.00 kcal/(mol Å [2]) connect pairs of nodes separated no more than 15 Å (the cutoff distance, denoted as *r*_*c*_) [38].

A Hessian matrix, reflecting small structural displacements around the energetic minimum on the potential energy surface, was constructed by computing the second derivative of the potential energy with respect to the Cα node coordinates [64]. Diagonalizing the Hessian matrix in ProDy yields eigenvectors (normal modes), which describe collective atomic motions, and eigenvalues, which correspond to the frequencies or energies of these modes. We selected the top 10 eigenvectors to calculate cross‐correlation matrices, which represent the correlation of movement patterns between all residue pairs within proteins.

### 2.2 Tiling Protocol and Dynamical Similarity Measures

Ferredoxin sequences were divided into contiguous subsequence tiles using a sliding‐window approach implemented in a custom Python script (see supplemental code and Figure S1). Each tile has a length *L*, where *L* ranges from six amino acids up to the full protein length *N*. The lower limit of six amino acids follows previous structure‐tiling work, where this length was shown to detect key structural motifs while maximizing sequence coverage [61]. To generate the complete set of tiles for a protein of length *N*, we consider each integer *L* in the interval 6 ≤ *L* ≤ *N*. For a specific length *L*, the *i*‐th tile *T*_i_ spans a contiguous subsequence of length *L*. Because we shift this window of length *L* across the entire protein, i (the tile index) ranges from 1 to (*N*–*L* + 1). Consequently, the total number of tiles *N*_t_ can be computed as:

(1) $$N_{t}=\sum_{L=6}^{N}\left(N-L+1\right)$$

**FIGURE 2:**
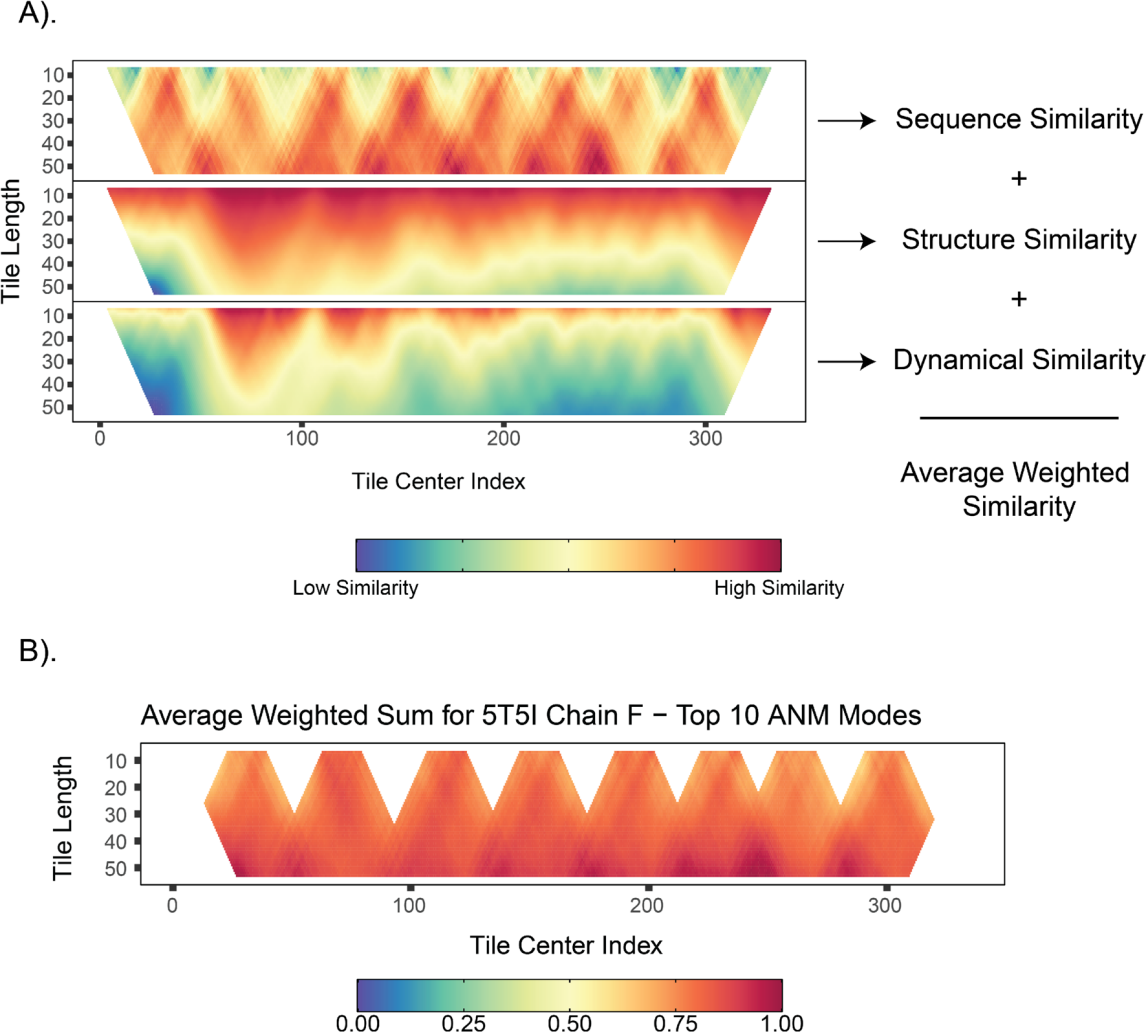
Overview of the methodology. (A) Depicts the three primary parameters (sequence similarity, structure similarity, dynamical similarity) and their distributions across different tile lengths and center indices. (B) The weighted sum for each element is taken as the sum of the product of the weights with each parameter. With the weighted sums calculated, the tiles are iterated over again and checked for the presence of any cysteines. If there are cysteines within an established bonding range to irons within an iron–sulfur cluster, then that tile is kept. If not, then they are discarded. This provided us with the final saw‐tooth pattern. Each of the “blades” of the sawtooth are designated as an “island,” and borders between any two islands are blocked. This is to constrain the search area when calculating the optimal path via the dynamic programming algorithm.

After generating every possible tile *T*_i_ of length *L*, we form all possible pairwise comparisons between tiles. We maintain a direct mapping between each amino acid's position in a tile and its residue index in the original structure. This residue‐based mapping allows us to retrieve the corresponding rows and columns in a precomputed cross‐correlation matrix, denoted as *C*. Specifically, for each pair of tiles *T*_i_ and *T*_j_ (both of length *L*), the indices of the amino acids in those tiles are used to extract a square submatrix *C*_ij_ containing only the cross‐correlations among residues in *T*_i_ and *T*_j_. These submatrices capture both the spatial arrangement of residues and their dynamical couplings as encoded in the full correlation matrix. Each submatrix is then flattened into two vectors ***u***_***i***_ (corresponding to *T*_i_) and ***v***_***j***_ (corresponding to *T*_j_). Once ***u***_***i***_ and ***v***_***j***_ are obtained, the cosine similarity (Figure 2A) between the two vectors measures their directional alignment. Mathematically, this is defined as:

(2) $$\cos\left(u_{i},v_{j}\right)=\frac{u_{i}\cdot v_{j}}{\left\|u_{i}\right\|\;\left\|v_{j}\right\|}$$

where ***u***_***i***_ and ***v***_***j***_ is the dot product of the two vectors and ||·|| denotes the Euclidean norm. The cosine similarity ranges from −1 (perfectly opposite orientations) to 1 (identical orientations), with 0 indicating orthogonal vectors.

In addition to assessing dynamical similarity via cosine similarity, we also measure the pairwise sequence similarity between any two tiles (Figure 2A). These calculations use the Smith‐Waterman algorithm from the Bio.Align sub‐package in the Biopython API [65], with the BLOSUM62 substitution matrix and default gap‐penalty parameters. The raw alignment score is divided by the average of the two tiles' maximum possible alignment scores, yielding a normalized similarity percentage for each comparison.

Lastly, to quantify structural differences between two tiles, we compute the Frobenius distance of their respective submatrices (Figure 2A). If **Q** represents the query tile's submatrix and **T** is the target tile's submatrix, both of size m × m, we first define a difference matrix **D** = **Q**–**T**. The Frobenius norm of D is then computed as:

(3) $${\left\|Q-T\right\|}_{F}={\left\|D\right\|}_{F}=\sqrt{\left\langle D,D\right\rangle }$$

The final portion of the expression yields us with the matrix inner product denoted as ⟨**D**, **D**⟩. Because the Frobenius norm is induced by a natural matrix inner product, we can reduce the above expression to the following:

(4) $${\left\|D\right\|}_{F}=\sqrt{\left\langle D,D\right\rangle }=\sqrt{\sum_{i}\sum_{j}D_{\mathrm{ij}}^{2}}$$

Substituting **Q**–**T** for **D** gives:

(5) $${\left\|D\right\|}_{F}=\sqrt{\left\langle Q,T\right\rangle }=\sqrt{\sum_{i}\sum_{j}{\left(Q_{\mathrm{ij}}-T_{\mathrm{ij}}\right)}^{2}}$$

where ***Q***_***ij***_ and ***T***_***ij***_ are the (*i*,*j*)‐th entries of matrices ***Q*** and ***T***, respectively. The resulting Frobenius distance is a single scalar value representing how dissimilar the two submatrices are in terms of their structural arrangement. Because every comparison involves two tiles of the same length *L*, the residue indices that define *T*_i_ and *T*_j_ map onto sub‐matrices of identical dimension (*m* = *L*). This equality is an intrinsic consequence of the sliding‐window approach. As a result, the Frobenius distance is therefore computed directly on **D** without any rigid‐body rotation or translation; the correlation coefficients are already invariant to such motions (Figure S2). Smaller Frobenius distances indicate greater similarity between the residue‐pair coupling between the two tiles, whereas larger values signify major differences.

However, since the Frobenius distance is a metric denoting dissimilarity, we must convert it into a bounded similarity before combing it with cosine and Smith–Waterman scores. Following the continuous form of the Gower's similarity coefficient [66], the transformation is

(6) $$S_{\text{Frobenius}}=1-\frac{d_{\text{Frobenius}}}{d_{\text{Frobenius},\max}}$$

Where d_Frobenius,max_ is the largest Frobenius distance observed across all tile pairs in the dataset. The mapping is affine and strictly monotonic, so it preserves rank order while aligning the direction of the metric with the two similarity‐based scores. After this step, all correlations can now be averaged coherently via the Bayesian objective function in Equation (9).

### 2.3 Determination of Optimal Weights Using Bayesian Optimization

Bayesian optimization was employed to objectively determine the best weights for each of the similarity metrics–cosine similarity, the Frobenius distance, and sequence similarity—so that a combined, weighted similarity measure would align closely with each of the individual metrics. Mathematically, for a given query *x*, the weighted similarity *S*_*weighted*_ *(x)* can be expressed as:

(7) $$S_{\text{weighted}}\left(x\right)=w_{\text{cosine}}\times S_{\text{cosine}}\left(x\right)+w_{\text{frobenius}}\times S_{\text{frobenius}}\left(x\right)+w_{\mathrm{sw}}\times S_{\mathrm{sw}}\left(x\right)$$

where each *S*_*metric*_ *(x)* (metric ∈ [cosine, Frobenius, SW]) denotes the respective similarity score, and *w*_*metric*_ is the weight assigned to that score. The subscript “SW” refers to sequence similarity obtained via the Smith–Waterman (*S*_sw_) algorithm.

To quantify how well *S*_weighted_(x) correlates with each individual similarity measure, we define three separate correlations:

$$C_{\text{cosine}}=\text{corr}\left(S_{\text{weighted}},S_{\text{cosine}}\right)$$

$$C_{\mathrm{sw}}=\text{corr}\left(S_{\text{weighted}},S_{\mathrm{sw}}\right)$$

(8) $$C_{\text{frobenius}}=\text{corr}\left(S_{\text{weighted}},S_{\text{frobenius}}\right)$$

where corr(*A, B*) denotes the statistical correlation between two sets of values *A* and *B*. The objective function O to be maximized, is given by the average of these three individual correlations:

(9) $$O\left(w_{\text{cosine}},w_{\text{frobenius}},w_{\mathrm{sw}}\right)=\frac{1}{3}\left(C_{\text{cosine}}+C_{\text{frobenius}}+C_{\mathrm{sw}}\right)$$

A Gaussian Process (GP) model was used to approximate the unknown objective function O [67]. By definition, a GP is fully specified by its mean function *m*(*x*) and covariance function *k*(*x,x*′). For simplicity, a constant value of 0 was chosen for the mean function, while the covariance was modeled by the squared exponential kernel:

(10) $$k\left(x,x^{\prime }\right)=\sigma^{2}\exp\left(-\frac{{\left(x-x^{\prime }\right)}^{2}}{2l^{2}}\right)$$

where 𝜎 represents the signal variance, and 𝘭 is the length‐scale parameter controlling the smoothness of the function. Once this GP was established, the Expected Improvement (EI) criterion was employed as the acquisition function, guiding the search for optimal weight values by balancing exploration and exploitation. The EI is defined as:

(11) $$\mathrm{EI}\left(x\right)=E\left[\max\left(f\left(x^{*}\right)-f\left(x\right),0\right)\right]$$

where *f*(x*) is the best observed value of the objective function up to the current iteration, and *f*(*x*) is the model's predicted value at a new point *x*.

In this study, the Bayesian optimization procedure was run for 115 iterations. The initial 15 iterations randomly sampled different weight combinations to build an initial training set for the GP model. The remaining 100 iterations were guided by the acquisition function, selecting new points *x* (i.e., sets the new weights) that were most likely to improve upon the best current solution. At the conclusion of this process, the procedure yielded a set of optimal, normalized weights (*w*_*cosine*_, *w*_*frobenius*_, *w*_*SW*_) for each query‐target structure pair. Notably, these weights were consistent across different structures in our data set, indicating that the relative contributions of the individual metrics remained stable. The final weighted similarity S_weighted_(*x*) computed with these optimal weights was then used to determine the most favorable pathways through the islands under investigation.

### 2.4 Computing the Optimal Paths for Advantageous Tile Sequences

The algorithm presented here identifies a sequence of tile lengths and tile center indices—collectively referred to as the optimal path—by maximizing a weighted sum encoded within a structure matrix called the *IslandTileMatrix*. In this matrix, the row index 𝘪 corresponds to a discrete tile length, and the column index *j* corresponds to a tile center index. Each entry in the matrix therefore represents the contribution (or weight) of adopting a specific tile length 𝘪 at a specific tile center index *j*. Larger tile lengths generally capture more of the underlying signal and are considered advantageous, but they can potentially obscure more localized dynamics. As a result, the algorithm seeks to balance these competing effects while identifying the path through the matrix that yields the greatest cumulative weight.

To accomplish this, two supplementary matrices of the same dimension as the IslandTileMatrix are maintained throughout the algorithm: the *dynamic programming matrix (DPM)* and the *traceback matrix (TM)*. The *DPM* is initialized such that its first row, 𝘪 = 0, receives values directly from the *IslandTileMatrix*. This initialization step enforces a defined starting state for the algorithm, although it is possible to restrict the range of valid tile center indices *j* if the application requires a narrower starting configuration. Formally, the initial condition for the DPM in Equation (12) is expressed as:

(12) $${\mathrm{DPM}}_{i,j}=\left\{\begin{array}{c} \;{\text{IslandTileMatrix}}_{i,j},\;\text{if}\;i=0\\ -\infty ,\;\text{otherwise} \end{array}\right.$$

As the algorithm advances row by row (i.e., by increasing 𝘪), each grid point DPM_*ij*_, is updated using the maximum value from the set of neighboring columns in the previous row. Specifically, for a cell indexed by (𝘪, *j*), the algorithm looks up to two columns on each side—namely (𝘪 − 1, *j* − 2), (𝘪 − 1, *j* − 1), (𝘪 − 1, *j*), (𝘪 − 1, *j* + 1), (𝘪 − 1, *j* + 2)—in the preceding row 𝘪 − 1. It then adds the corresponding *IslandTileMatrix* value, IslandTileMatrix_ij_, to the maximum among those potential predecessors. This process can be expressed as the recurrence relation in Equation (13):

(13) $$\begin{array}{c} {\mathrm{DPM}}_{i,j}={\text{IslandTileMatrix}}_{i,j}+\max({\mathrm{DPM}}_{i-1,j-2},{\mathrm{DPM}}_{i-1,j-1},\\ \;{\mathrm{DPM}}_{i-1,j},{\mathrm{DPM}}_{i-1,j+1},{\mathrm{DPM}}_{i-1,j+2}) \end{array}$$

At the same time, the *TM* stores the column index *ĵ* that produces this maximum value for each (𝘪, *j*). Once the algorithm has filled all rows of the *DPM*, the optimal path is reconstructed by locating the maximum entry in the final row and then iteratively backtracking through the *TM*. The resulting optimal path comprises an ordered list of (𝘪, *j*) pairs—each pair containing a tile length and a tile center index—that capture the globally optimal traversal through the *IslandTileMatrix*. This set of pairs can be denoted as follows:

(14) $$\text{optimal}\_\text{path}=\left\{\left(i_{1},j_{1}\right),\left(i_{2},j_{2}\right),\ldots ,\left(i_{m},j_{m}\right)\right\}$$

Upon completing the traceback, the final sequence of tile lengths and tile center indices reflects the best balance between choosing larger tiles to capture more extensive signal and sufficiently small tiles to preserve the underlying local dynamics. All detailed implementation steps for this procedure are provided in the Supporting Information labeled “OptimalTilePathAlgorithm_Version1.0.py.”

### 2.5 Ranking and Mapping Isolated Tiles Back Onto Protein Structure

All three‐similarity metrics used in this study were ranked and then summed to form a single composite score (Figure 2B). Let *N* denote the total length of the protein structure under examination, and let each tile have length *L*, where *6 ≤ L ≤ N/2*. Each tile in this length range was sorted in ascending order according to its composite score, and any tiles whose cosine similarity or sequence similarity fell below 50% was discarded. From the remaining candidates, the top 20% were written to temporary files, and once the tile length reached *N/2*, all of these partial outputs were concatenated into a single dataset. A subsequent refinement step then excluded tiles if they lacked cysteine residues altogether or if the spatial positions of those cysteine residues were inconsistent with typical iron–sulfur (Fe–S) bonding distances, specifically lying outside the range 2.18 Å ≤ *r* ≤ 2.35 Å [68, 69]. This final filtering measure helps avoid spurious matches in structurally unrelated motifs, such as Rossmann‐like folds or other β–α–β super‐secondary structures. Since each tile's residue indices map directly to those in the corresponding PDB file, the method produces a PyMOL script that highlights all identified matches on the protein structure, enabling straightforward visualization of these tiles in a three‐dimensional context.

## 3 Results and Discussion

### 3.1 Query and Target Selection

To identify ferredoxin subdomains within larger metalloproteins, we obtained structures and sequences from the Protein Data Bank (rcsb.org). The query structure was the 2[4Fe‐4S] ferredoxin from *G*. *acidurici* (PDB: 2FDN, *N* = 55) [60]. As target structures, we selected three larger metalloproteins: a formyl‐methanofuran dehydrogenase (Fwd) from *Methanothermobacter wolfeii* (PDB: 5T5I, *N* = 3622) [70], dihydropyrimidine dehydrogenase (DPD) from *Sus scrofa* (PDB: 1GTE, *N* = 2010) [71], and a methanogenic heterodisulfide reductase from *Methanothermococcus thermolithotrophicus* (PDB: 5ODC, *N* = 4011) [72]. Previous structure‐based approaches identified common ferredoxin‐like domains in Fwd, DPD, and the heterodisulfide reductase [18]. Furthermore, DPD and the heterodisulfide reductase contain variant iron–sulfur folds that share low structural similarity with ferredoxin.

For each query/target pair, we computed cross‐correlation matrices using the coarse‐grained ANM as implemented in ProDy [38, 63]. Each cross‐correlation matrix is of dimension *N* × *N*, where *N* denotes the total number of residues in the structure being analyzed. The entry in the 𝘪‐th row and *j*‐th column of this matrix reflects the dynamical correlation between the 𝘪‐th and *j*‐th residues. When generating a tile of length *L* from a query sequence, the residue indices in that tile map directly to the corresponding residue indices in the *N* × *N* cross‐correlation matrix, allowing for precise extraction of a relevant submatrix. This one‐to‐one mapping ensures that the subset of the matrix fully corresponds to the positions of amino acids in both sequence and structure. Dynamical and structural similarity between each query tile and target tiles in the larger protein was subsequently evaluated using two complementary metrics: cosine similarity and a Frobenius‐distance‐derived similarity score. By comparing these submatrices across sliding‐window tiles, the algorithm uncovered correspondences that highlight local ferredoxin‐like subdomains within broader protein folds.

### 3.2 Tile Length Selection in Dynamical Similarity Detection

The choice of tile length, denoted here as *L*, is critical for capturing the most informative range of local and global motions in dynamical similarity analyses. Shorter tiles provide higher resolution of localized dynamics but risk overlooking broader global features. Conversely, longer tiles resolve larger‐scale patterns, but they do so at the possible expense of losing key local motions. Balancing these two considerations allows for both sensitivity (the likelihood of detecting meaningful local correlations) and specificity (the avoidance of spurious matches driven by overly large tiles).

A similar dilemma has long been recognized in sequence alignments. Early heuristic programs such as FASTA introduced the idea of scanning for short k‐tuple “words,” and Altschul et al. [73] later embedded a tunable word size W in the BLAST algorithm to balance speed and sensitivity. They showed that shorter words increase sensitivity at a computational cost, whereas longer words accelerate searches but can miss important alignments. Although BLAST originally proposed a word size of four residues as a compromise [73], subsequent studies suggest that windows of 10 and 20 residues best preserve local amino acid composition while minimizing statistical noise in homology detection [74].

Likewise, in structural analyses, Parra et al. [61] pioneered a tiling approach and recommended a starting tile length of six. Tiles shorter than six residues were deemed too small for meaningful structural comparison based on alpha carbon traces, while a tile length of at least six effectively captures common structural elements such as beta strands (4–6 residues) and smaller alpha helices (6–40 residues) [75, 76]. Within this study, we retained this lower bound of *L* = 6 for identifying subdomains, but our upper bound was naturally set by the length of the query protein, *N* = 55. As a result, the largest tile considered spans the entire query (i.e., 55 residues), with different tile center indices explored along the length of the target proteins.

To determine the most suitable tile lengths for detecting meaningful dynamics, we performed a polynomial‐based non‐linear regression on the average weighted sum of our three‐similarity metrics (cosine similarity, sequence similarity, and Frobenius distance) as a function of *L*. We denote this function as *S*(*L*). By examining the derivative of *S′*(*L*), we identified points where changes in the average weighted sum stabilized, implying a balance between capturing local detail and broader motion. To enhance the detection of these stabilization points, the derivative was first smoothed with a Gaussian filter and then subjected to a rolling average. The optimal tile length for each path was taken as the smallest *L* for which the smoothed derivative approached zero, indicating diminishing returns in the average weighted sum. Because different paths—defined by their unique sequence and structural contexts—may converge at different rates, this approach was applied on a per‐path basis rather than relying on a single global threshold. An example illustrating this derivative‐based convergence in the formyl‐methanofuran dehydrogenase (Fwd) data set is provided in (Figure S3).

### 3.3 A Poly‐Ferredoxin Target

We considered the Fwd domain as a positive control for the comparative dynamics method (Figure S4). Fwd has 12 subunits—a dimer of heterohexamers (FwdABCDEFG). Of particular interest are the *F* and *G* subunits. These two subunits form an electron‐supplying core, with the FwdF dimer acting like a wire [70]. The FwdF subunit is a frank polyferredoxin with four ferredoxin domains binding a total of eight [4Fe–4S] clusters. This would be relatively straightforward to detect even by sequence or structure‐based methods. In contrast, the FwdG subunit contains one ferredoxin domain binding 2[4Fe–4S] clusters [70, 77, 78, 79], with a topology that diverges from the canonical fold (β–α–β half + α‐helical half). Additional iron–sulfur clusters exist in the dodecamer—such as in FwdB.

**FIGURE 3:**
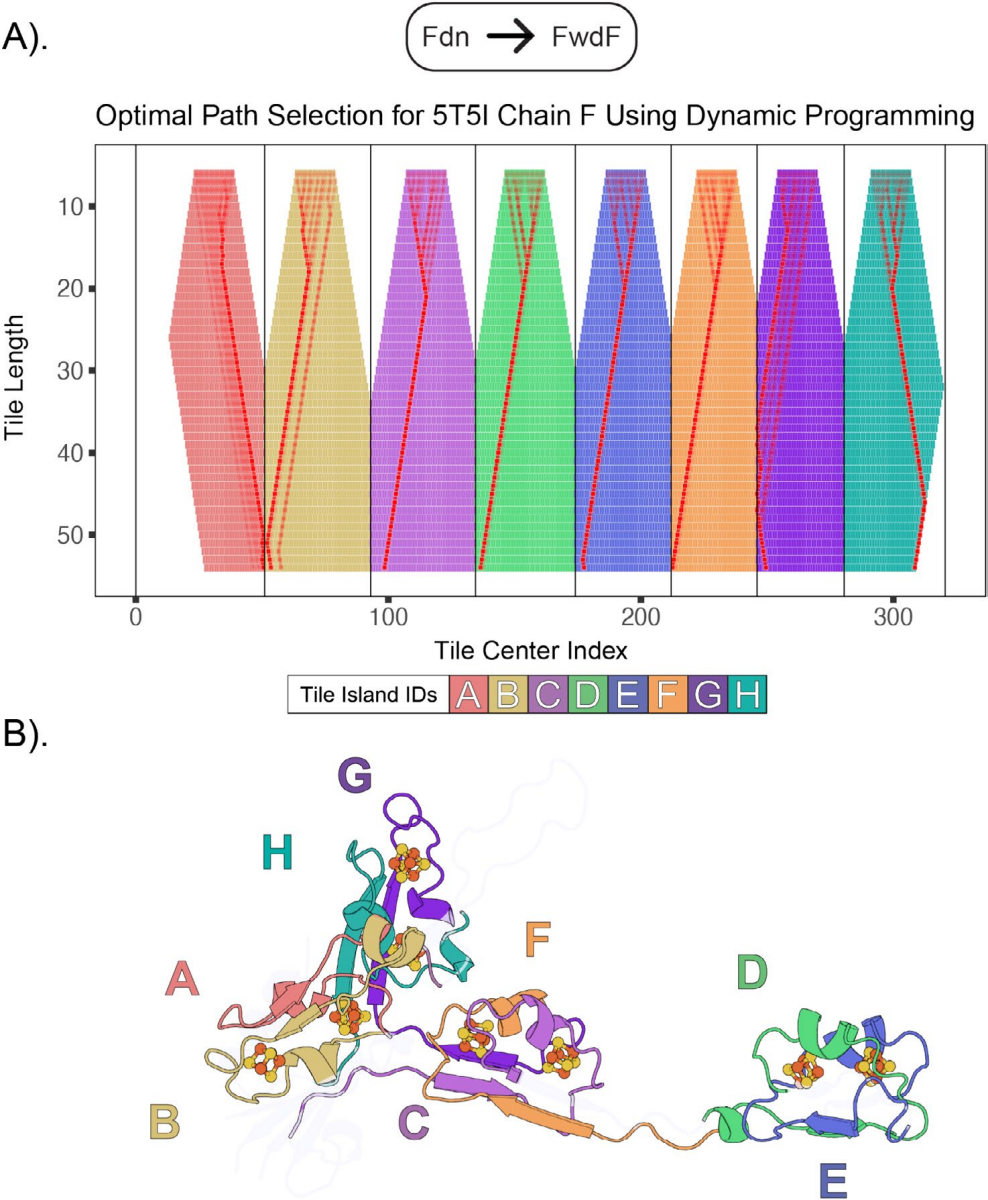
Ferredoxin domains in the FwdF subunit. The optimal path, as determined by the dynamic programming algorithm, is illustrated above via the red lines and squares in (A). The tile length and center index combinations along this path represent portions of the original structure that map back onto it, colored according to their tile island ID, as shown in (B). These optimal matches correspond to all of the ferredoxin halves contained within the subunit.

FwdF is a positive control with a previously described polyferredoxin fold [70]. After filtering for cysteines within bonding range of an iron–sulfur cluster in FwdF, we observe eight separate islands consistent with the original structural study detailing four embedded ferredoxin domains binding eight [4Fe–4S] clusters [70, 77, 78, 79]. With the exception of islands B, F, and G, the clear structural homology of the embedded domains to the β–α–β query allowed rapid convergence on optimal tile lengths and positions for each of the islands (Figure 3).

**FIGURE 4:**
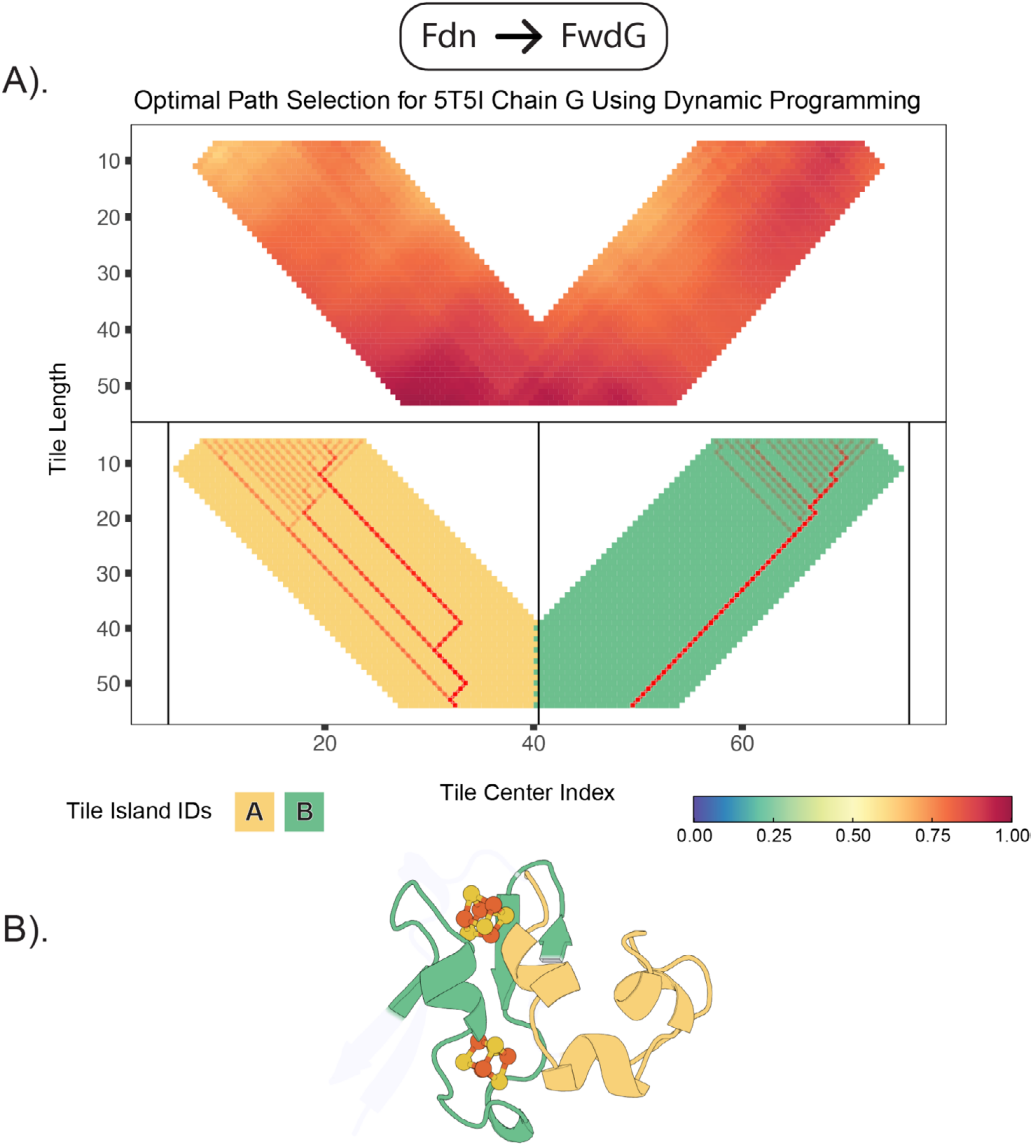
Ferredoxin domains in the FwdG subunit. After calculating the weighted sum and applying the cysteine‐iron bond length constraints in (A, top), we're left with two distinct peaks which represent each half ferredoxin half. In (A, bottom), this is used as input for the dynamic programming algorithm which constructs the optimal path by maximizing the values of the weighted sum along each step. The highlighted portions of the fold in (B) are those sequences selected by the algorithm which have satisfied that requirement.

Two distinct islands (A and B) were observed in the FwdG tiling plot (Figure 4A). Both share dynamics, structure, and sequence with the β–α–β half of a bacterial ferredoxin. This is perhaps surprising, given that island A corresponds to an all alpha‐helical fold (Figure 4B). This is reflected in the optimum path through the island. Island A contains more branching paths than island B. These multiple paths at shorter tile lengths indicate local similarity between the query and target. Convergence at longer tile lengths suggests an optimal global match between the helical target and the β–α–β query. This indicates that comparative dynamics can sometimes supersede lack of clear sequence or structural homology.

### 3.4 Ferredoxin Subdomains in DPD

**FIGURE 5:**
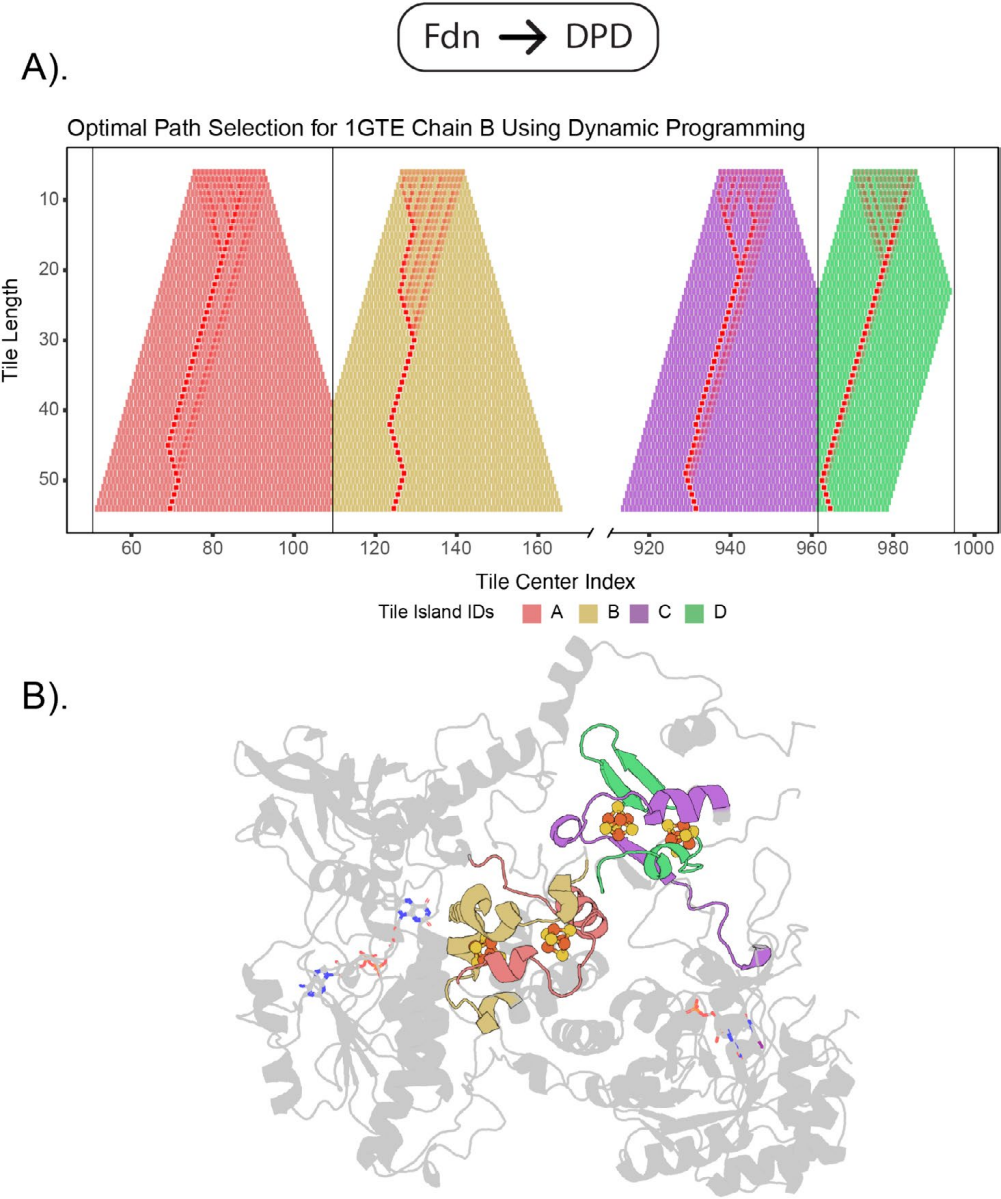
Ferredoxin domains in Chain A of DPD. After filtering the weighted sums with the cysteine‐iron bond length constraints, only four peaks remain. Each peak here references a ferredoxin half. Optimal matches obtained in (A) and mapped onto the structure in (B) pinpoint the ferredoxin domains precisely in DPD.

DPD chain A contains multiple iron–sulfur binding domains, some of which are all‐helical instead of the typical β–α–β fold. Four islands are detected. Islands A and B correspond to two halves of the α‐helical domain (from position 50 to 162), and islands C and D correspond to the β–α–β fold (position 934 to 992) (Figure 5A). Optimal paths converge quickly around a tile length of 20, with the exception being island B, which fully converges around a tile length of 30. Interestingly, the optimal path for island D appears to converge to the boundary separating the two islands—indicating the best match to the query is a full 2(β–α–β) fold (Figure 5B)—whereas the optimal paths for islands A and B cover the entirety of the full α‐helical domain.

These non‐canonical ferredoxins are found in many other oxidoreductases. Pyruvate: ferredoxin oxidoreductase (PFOR) contains a 2(β–α–β) bacterial ferredoxin and an all‐helical quinol‐fumarate reductase ferredoxin in distinct regions of the protein [80]. Despite the structural differences, sequence similarity between the two ferredoxin domains in PFOR are substantial [80]. Instances such as this highlight the importance and utility behind using multiple similarity metrics for sequence, structure, and dynamics in comparative domain detection.

### 3.5 Ferredoxin Subdomains in the HdrABC–MvhAGD Complex

**FIGURE 6:**
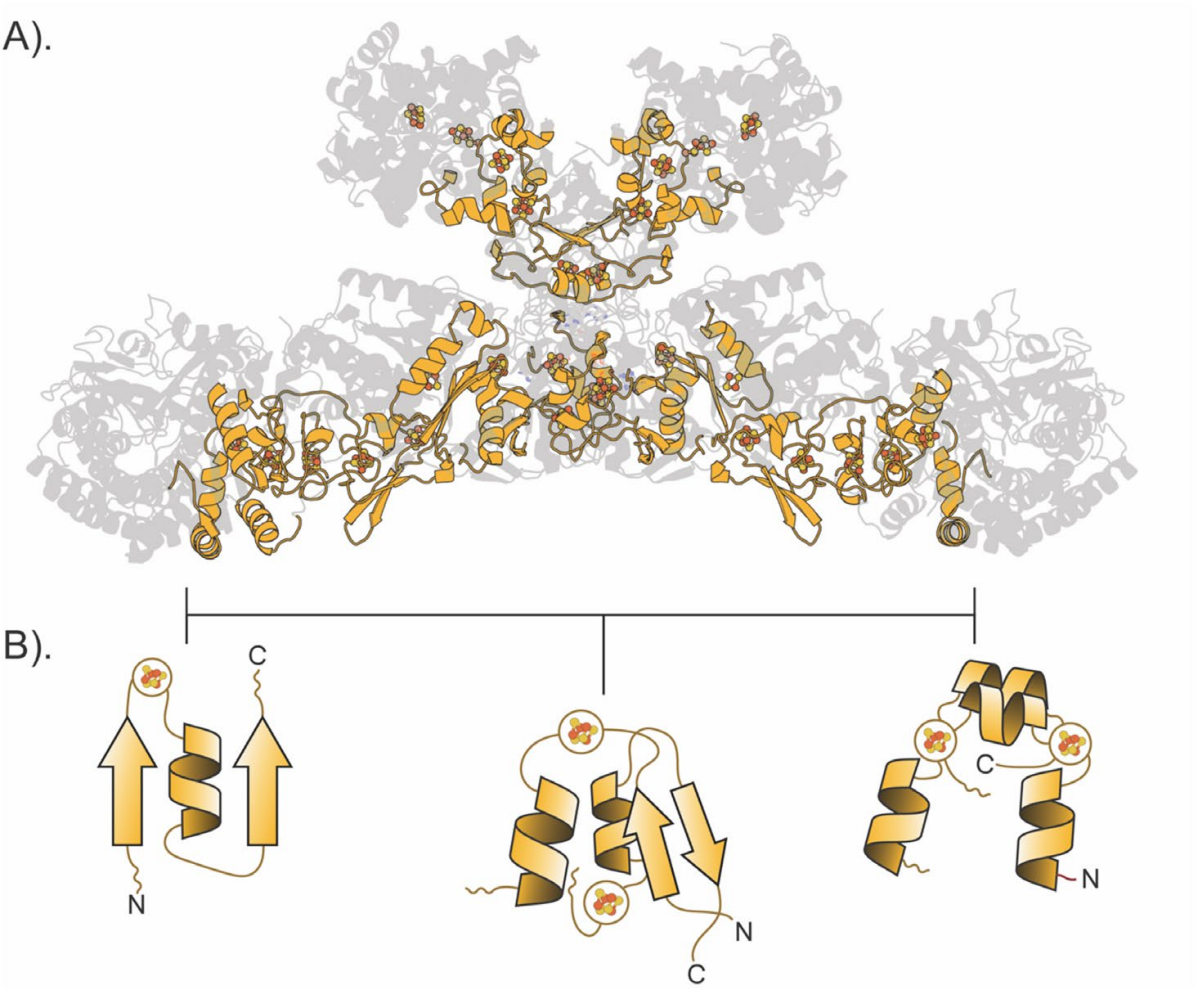
Optimal matches mapped onto HdrABC‐MvhAGD. (A) Ferredoxin domains in the expansive heterodisulfide reductase are outlined here in yellow. (B) Ferredoxin fold variety within the structure depicted as a cartoon. Domains here comprise of half ferredoxins (1xβαβ), truncated half‐ferredoxin pairs (1–2xβα), and all α‐helical ferredoxins.

The heterodisulfide reductase‐NiFe hydrogenase linked enzyme (HdrABC–MvhAGD), found in the cytoplasm of most methanogenic archaea [81], is essential for the first step of methanogenesis. It reduces the heterodisulfide of coenzymes M, B, and ferredoxin through the oxidation of H_2_ [82]. The complex contains a multitude of bound iron–sulfur clusters of varying stoichiometries, including a non‐cubane [4Fe–4S] cluster and a [2Fe–2S] cluster. Further, the topologies of these ferredoxins are also varied, ranging from canonical half‐ferredoxins to all α‐helical ferredoxins (Figure 6B). These different [Fe–S] clusters all use cysteine as the first‐shell ligand and would be selected in the initial filtering step. This tests whether the dynamics‐based approach can identify [4Fe–4S] binding ferredoxins.

The structure of Hdr is larger and more complex than the previous targets. Still, despite that, the algorithm quickly converged on several [4Fe–4S] ferredoxin domains (Figure 6A and Figure S5). No matches were found with [2Fe–2S] ferredoxin domains, suggesting that these plant‐type ferredoxins do not match bacterial ferredoxins at the sequence, structure, or dynamical level. Despite structural variations between corresponding chains of the two heterohexamers, the same tiles were identified.

**FIGURE 7:**
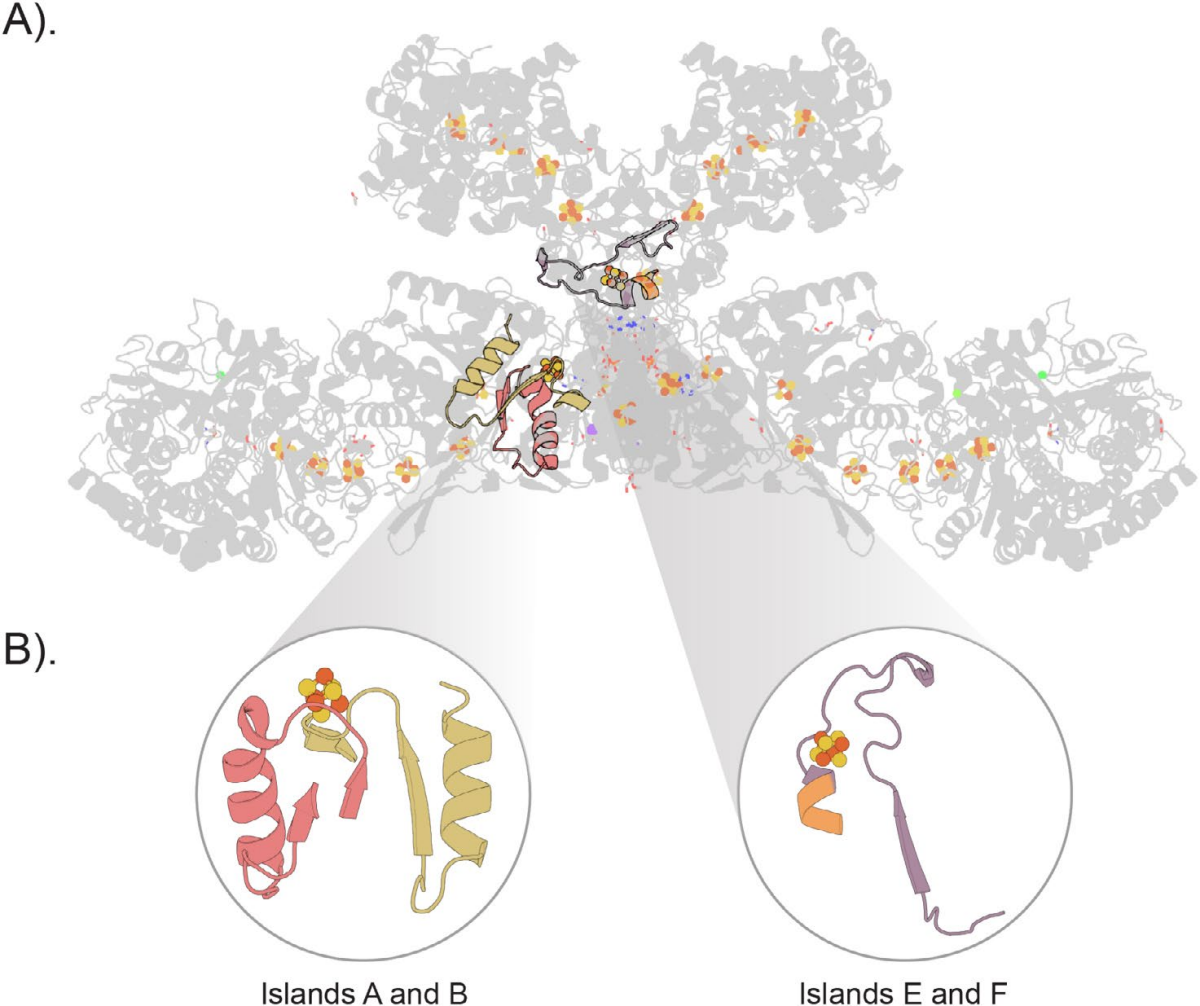
Optimal matches within four islands in HdrG depict mixed Rossmann and Ferredoxin Domains. (A) Optimal matches from Islands A, B, E, and F are highlighted within the context of the greater structure. (B) Optimal matches from these islands are magnified and look like half‐ferredoxins that have been inserted into Rossmann Folds.

In chains A and G, we find that the ferredoxin query maps onto portions of two Rossmann folds of Hdr, each binding one [4Fe–4S] cluster (Figure 7A,B). This observation is consistent with previous analyses suggesting a common ancestor to ferredoxin and Rossmann‐like folds [18]. The fact that these areas exhibit dynamical overlap, in addition to sequence and structural similarity, supports the hypothesis of shared ancestry. This also highlights that functionally similar units, as determined by their dynamic properties, can be used to infer homology.

### 3.6 Dynamical vs. Structural Similarity

**FIGURE 8:**
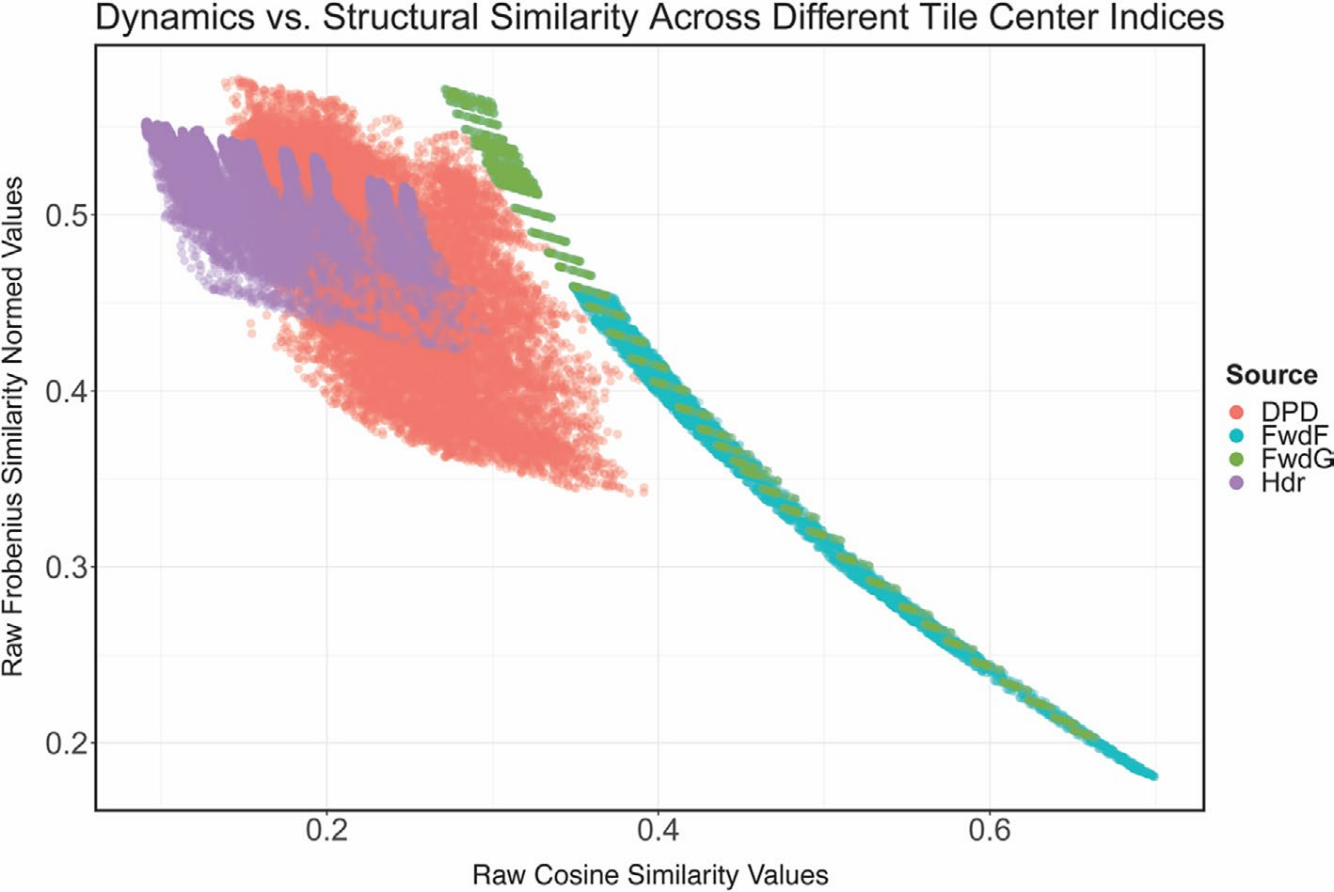
Dynamical and structural similarity metrics exhibit a monotonic, curvilinear relationship. Cosine similarity is plotted against the Frobenius similarity across all tile center indices for tiles 30 residues in length. The distribution depicts a curvilinear relationship, with faster rates of change observed for cosine similarity at the lower right and for Frobenius similarity at the upper left. This pattern persists across each model's distribution, where an inflection point indicates a change in concavity, illustrating where structural similarity degrades at a rate that is incongruent with dynamical similarity.

This study rests on the premise that, among the three key factors—sequence, structure, and dynamics—dynamics is the most conserved over deep evolutionary time as it is most directly linked to protein function. From this standpoint, we hypothesize that overlapping dynamic behavior implies functional similarity and that the long‐term conservation of these dynamics may indicate homology between structural units. To assess this quantitatively, we compared the structural (Frobenius) similarity against dynamical (cosine) similarity for all query‐target tile pairs of *L* = 30 residues in length (Figure 8).

In the resulting plot, ferredoxin‐Fwd query‐target pairs concentrate in the lower‐right portion, where both structural and dynamical similarity scores are relatively high. This pattern reflects the clear repetition of β–ɑ–β subdomains that form part of the larger Fwd protein. The relationship between structure and dynamics appears to be nonlinear: as structural similarity degrades, the corresponding dynamical similarity remains comparatively robust, suggesting that a protein's functionally related motions can persist even when homology is less pronounced (Figure S6). Hdr and DPD show generally weak similarity on both axes but follow the same nonlinear trend, supporting the idea that the dynamical features of ferredoxins embedded within larger oxidoreductases are preserved over deep time. Finally, residue ranges for each of the islands and their respective structures are highlighted in (Table S1).

## 4 Conclusion

Of protein sequence, structure, and dynamics, the last is most directly connected to function, and therefore evolutionary selection. Dynamical information coupled with sequence and structural attributes can identify bacterial ferredoxin regions within larger proteins. We have been able to isolate ferredoxin folds in target structures that are topologically dissimilar to the query. We find embedded ferredoxin domains within larger proteins, including all α‐helical topologies and β–α–β motifs inside Rossmann folds, which supports the inference of homology derived from conserved functional dynamics.

The success of our method depends on the appropriate selection of tile length. When applying this method to domains embedded within other proteins, we ensure that the maximum permissible tile length is bounded by the length of the parent domain or input query structure. In this work, the query structure, a 2×[4Fe–4S] bacterial ferredoxin, remained consistent while the target structures varied. Therefore, the tile length was constrained to a range between a minimum of 6 residues and a maximum of 55 residues. However, heme‐binding cytochromes are also well characterized [83, 84], and include both small, soluble electron carriers and embedded cytochrome folds within larger enzymes and protein nanowires. Given the centrality of redox catalysis in the evolution of metabolism, we seek to improve upon and extend this approach to structures beyond ferredoxins, such as globin folds or cytochrome P450s, where their tile lengths would range from 6 to the maximum length of the parent domain.

Finally, while the dynamics captured by ANM are invaluable for understanding the large‐scale, collective motions of proteins, it is important to recognize its inherent limitations. This model is based on NMA, which operates under the assumption that atomic displacements occur within a harmonic potential. This assumption restricts its ability to explore the full potential energy surface (PES). As a result, ANM primarily samples the immediate vicinity around the equilibrium state, leaving out more complex, functionally relevant motions that may occur in regions of the PES characterized by anharmonicity.

Anharmonic modes encompass movements that extend beyond this harmonic approximation and involve navigating broader, more complex areas of the PES. These areas include features such as energy barriers, non‐linear paths, and multiple local minima. It has been shown that ANM, when complemented by MD simulations, can capture a greater range of functionally relevant dynamics [49]. Furthermore, previous work has enhanced NMA by incorporating anharmonic aspects, sampling an anharmonic potential function along the directions of the eigenvectors of the lowest normal modes [85].

In this work, we have used classic ANM for our dynamics calculations because many of the structures are too large and contain metals that require specific parameters to model accurately. However, it would be worthwhile in future work to consider the impact of anharmonicity on homology detection via methods such as ANMA. Doing so would provide us with a more comprehensive understanding of the dynamics and improve the prediction of functionally relevant conformational changes.

## Author Contributions

**Jan A. Siess:** conceptualization, data curation, formal analysis, investigation, methodology, software, validation, writing – original draft, writing – review and editing, visualization. **Vikas Nanda:** conceptualization, methodology, project administration, resources, writing – original draft, writing – review and editing, funding acquisition, investigation, supervision.

## Conflicts of Interest

The authors declare no conflicts of interest.

## Peer Review

The peer review history for this article is available at https://www.webofscience.com/api/gateway/wos/peer‐review/10.1002/prot.70004.

## Supporting information


**Data S1.** Supporting Information.

## Data Availability

The data that support the findings of this study are openly available in ComparativeDynamics_Code‐v1.0.0 at https://doi.org/10.5281/zenodo.11099660, reference number 10.5281/zenodo.11099661.

## References

[1] J. E. Goldford and D. Segrè , “Modern Views of Ancient Metabolic Networks,” Current Opinion in Systems Biology 8 (2018): 117–124.

[2] M. Bashton and C. Chothia , “The Generation of New Protein Functions by the Combination of Domains,” Structure 15, no. 1 (2007): 85–99, 10.1016/j.str.2006.11.009. 17223535

[3] J. A. Gerlt and P. C. Babbitt , “Divergent Evolution of Enzymatic Function: Mechanistically Diverse Superfamilies and Functionally Distinct Suprafamilies,” Annual Review of Biochemistry 70 (2001): 209–246, 10.1146/annurev.biochem.70.1.209. 11395407

[4] F. Baymann , E. Lebrun , M. Brugna , B. Schoepp‐Cothenet , M. T. Giudici‐Orticoni , and W. Nitschke , “The Redox Protein Construction Kit: Pre‐Last Universal Common Ancestor Evolution of Energy‐Conserving Enzymes,” Philosophical Transactions of the Royal Society of London. Series B, Biological Sciences 358, no. 1429 (2003): 267–274, 10.1098/rstb.2002.1184. 12594934 PMC1693098

[5] A. K. Garcia , D. F. Harris , A. J. Rivier , et al., “Nitrogenase Resurrection and the Evolution of a Singular Enzymatic Mechanism,” eLife 12 (2023): 1–18, 10.7554/eLife.85003. PMC9977276 36799917

[6] A. K. Garcia , B. Kolaczkowski , and B. Kaçar , “Reconstruction of Nitrogenase Predecessors Suggests Origin From Maturase‐Like Proteins,” Genome Biology and Evolution 14, no. 3 (2022): evac031. 35179578 10.1093/gbe/evac031 PMC8890362

[7] S. L. Schwartz , A. K. Garcia , B. Kaçar , and G. P. Fournier , “Early Nitrogenase Ancestors Encompassed Novel Active Site Diversity,” Molecular Biology and Evolution 39, no. 11 (2022): msac226. 36260513 10.1093/molbev/msac226 PMC9641968

[8] L. A. David and E. J. Alm , “Rapid Evolutionary Innovation During an Archaean Genetic Expansion,” Nature 469, no. 7328 (2011): 93–96. 21170026 10.1038/nature09649

[9] K. Mateos , G. Chappell , A. Klos , et al., “The Evolution and Spread of Sulfur Cycling Enzymes Reflect the Redox State of the Early Earth,” Science Advances 9, no. 27 (2023): eade4847. 37418533 10.1126/sciadv.ade4847 PMC10328410

[10] H. Decker and K. E. Van Holde , Oxygen and the Evolution of Life (Springer Science & Business Media, 2010).

[11] P. G. Falkowski , “From Light to Life,” Origins of Life and Evolution of the Biosphere 45, no. 3 (2015): 347–350, 10.1007/s11084-015-9441-6. 26105723

[12] P. Kapli , T. Flouri , and M. J. Telford , “Systematic Errors in Phylogenetic Trees,” Current Biology 31, no. 2 (2021): R59–R64, 10.1016/j.cub.2020.11.043. 33497629

[13] K. Qiu , N. Ben‐Tal , and R. Kolodny , “Similar Protein Segments Shared Between Domains of Different Evolutionary Lineages,” Protein Science 31 (2022): e4407. 36040261 10.1002/pro.4407 PMC9387206

[14] R. Kolodny , S. Nepomnyachiy , D. S. Tawfik , and N. Ben‐Tal , “Bridging Themes: Short Protein Segments Found in Different Architectures,” Molecular Biology and Evolution 38, no. 6 (2021): 2191–2208. 33502503 10.1093/molbev/msab017 PMC8136508

[15] N. Ferruz , F. Lobos , D. Lemm , et al., “Identification and Analysis of Natural Building Blocks for Evolution‐Guided Fragment‐Based Protein Design,” Journal of Molecular Biology 432, no. 13 (2020): 3898–3914. 32330481 10.1016/j.jmb.2020.04.013 PMC7322520

[16] A. E. Todd , C. A. Orengo , and J. M. Thornton , “Evolution of Function in Protein Superfamilies, From a Structural Perspective,” Journal of Molecular Biology 307, no. 4 (2001): 1113–1143. 11286560 10.1006/jmbi.2001.4513

[17] H. Raanan , S. Poudel , D. H. Pike , V. Nanda , and P. G. Falkowski , “Small Protein Folds at the Root of an Ancient Metabolic Network,” National Academy of Sciences of the United States of America 117, no. 13 (2020): 7193–7199. 10.1073/pnas.1914982117 PMC7132300 32188785

[18] H. Raanan , D. H. Pike , E. K. Moore , P. G. Falkowski , and V. Nanda , “Modular Origins of Biological Electron Transfer Chains,” Proceedings of the National Academy of Sciences of the United States of America 115, no. 6 (2018): 1280–1285, 10.1073/pnas.1714225115. 29358375 PMC5819401

[19] J. Catazaro , A. Caprez , A. Guru , D. Swanson , and R. Powers , “Functional Evolution of PLP‐Dependent Enzymes Based on Active‐Site Structural Similarities,” Proteins: Structure, Function, and Bioinformatics 82, no. 10 (2014): 2597–2608. 10.1002/prot.24624 PMC4177364 24920327

[20] A. Narunsky , A. Kessel , R. Solan , V. Alva , R. Kolodny , and N. Ben‐Tal , “On the Evolution of Protein–Adenine Binding,” National Academy of Sciences of the United States of America 117, no. 9 (2020): 4701–4709. 10.1073/pnas.1911349117 PMC7060716 32079721

[21] S. Nepomnyachiy , N. Ben‐Tal , and R. Kolodny , “Complex Evolutionary Footprints Revealed in an Analysis of Reused Protein Segments of Diverse Lengths,” Proceedings of the National Academy of Sciences of the United States of America 114, no. 44 (2017): 11703–11708, 10.1073/pnas.1707642114. 29078314 PMC5676897

[22] L. A. Kelley and M. J. Sternberg , “Partial Protein Domains: Evolutionary Insights and Bioinformatics Challenges,” Genome Biology 16, no. 1 (2015): 100, 10.1186/s13059-015-0663-8. 25986583 PMC4436111

[23] I. Walsh , A. J. Martin , C. Mooney , E. Rubagotti , A. Vullo , and G. Pollastri , “Ab Initio and Homology Based Prediction of Protein Domains by Recursive Neural Networks,” BMC Bioinformatics 10 (2009): 195, 10.1186/1471-2105-10-195. 19558651 PMC2711945

[24] J. Eickholt , X. Deng , and J. Cheng , “DoBo: Protein Domain Boundary Prediction by Integrating Evolutionary Signals and Machine Learning,” BMC Bioinformatics 12, no. 43 (2011): 1–8, 10.1186/1471-2105-12-43. 21284866 PMC3036623

[25] K. F. Fischer and S. Marqusee , “A Rapid Test for Identification of Autonomous Folding Units in Proteins,” Journal of Molecular Biology 302, no. 3 (2000): 701–712. 10986128 10.1006/jmbi.2000.4049

[26] M. Varadi , S. Anyango , M. Deshpande , et al., “AlphaFold Protein Structure Database: Massively Expanding the Structural Coverage of Protein‐Sequence Space With High‐Accuracy Models,” Nucleic Acids Research 50, no. D1 (2022): D439–D444, 10.1093/nar/gkab1061. 34791371 PMC8728224

[27] J. Jumper , R. Evans , A. Pritzel , et al., “Highly Accurate Protein Structure Prediction With AlphaFold,” Nature 596, no. 7873 (2021): 583–589, 10.1038/s41586-021-03819-2. 34265844 PMC8371605

[28] Z. Lin , H. Akin , R. Rao , et al., “Evolutionary‐Scale Prediction of Atomic‐Level Protein Structure With a Language Model,” Science 379, no. 6637 (2023): 1123–1130, 10.1126/science.ade2574. 36927031

[29] R. Krishna , J. Wang , W. Ahern , et al., “Generalized Biomolecular Modeling and Design With RoseTTAFold All‐Atom,” Science 384, no. 6693 (2024): eadl2528, 10.1126/science.adl2528.38452047

[30] U. Hensen , T. Meyer , J. Haas , R. Rex , G. Vriend , and H. Grubmuller , “Exploring Protein Dynamics Space: The Dynasome as the Missing Link Between Protein Structure and Function,” PLoS One 7, no. 5 (2012): e33931, 10.1371/journal.pone.0033931. 22606222 PMC3350514

[31] Y. Wang , H. Zhang , H. Zhong , and Z. Xue , “Protein Domain Identification Methods and Online Resources,” Computational and Structural Biotechnology Journal 19 (2021): 1145–1153, 10.1016/j.csbj.2021.01.041. 33680357 PMC7895673

[32] C. Chothia and A. M. Lesk , “The Relation Between the Divergence of Sequence and Structure in Proteins,” EMBO Journal 5, no. 4 (1986): 823–826. 3709526 10.1002/j.1460-2075.1986.tb04288.x PMC1166865

[33] C. Sander and R. Schneider , “Database of Homology‐Derived Protein Structures and the Structural Meaning of Sequence Alignment,” Proteins 9, no. 1 (1991): 56–68, 10.1002/prot.340090107. 2017436

[34] K. Illergård , D. H. Ardell , and A. Elofsson , “Structure Is Three to Ten Times More Conserved Than Sequence—A Study of Structural Response in Protein Cores,” Proteins: Structure, Function, and Bioinformatics 77, no. 3 (2009): 499–508. 10.1002/prot.22458 19507241

[35] Y. Liu and I. Bahar , “Sequence Evolution Correlates With Structural Dynamics,” Molecular Biology and Evolution 29, no. 9 (2012): 2253–2263. 22427707 10.1093/molbev/mss097 PMC3424413

[36] C. L. Worth , S. Gong , and T. L. Blundell , “Structural and Functional Constraints in the Evolution of Protein Families,” Nature Reviews. Molecular Cell Biology 10, no. 10 (2009): 709–720, 10.1038/nrm2762. 19756040

[37] I. Bahar , A. R. Atilgan , M. C. Demirel , and B. Erman , “Vibrational Dynamics of Folded Proteins: Significance of Slow and Fast Motions in Relation to Function and Stability,” Physical Review Letters 80, no. 12 (1998): 2733.

[38] A. R. Atilgan , S. R. Durell , R. L. Jernigan , M. C. Demirel , O. Keskin , and I. Bahar , “Anisotropy of Fluctuation Dynamics of Proteins With an Elastic Network Model,” Biophysical Journal 80, no. 1 (2001): 505–515, 10.1016/S0006-3495(01)76033-X. 11159421 PMC1301252

[39] E. Braun , J. Gilmer , H. B. Mayes , et al., “Best Practices for Foundations in Molecular Simulations [Article v1.0],” Living Journal of Computational Molecular Science 1, no. 1 (2019): 5957. 31788666 10.33011/livecoms.1.1.5957 PMC6884151

[40] S. Piana , K. Lindorff‐Larsen , and D. E. Shaw , “Protein Folding Kinetics and Thermodynamics From Atomistic Simulation,” Proceedings of the National Academy of Sciences of the United States of America 109, no. 44 (2012): 17845–17850, 10.1073/pnas.1201811109. 22822217 PMC3497772

[41] J. Cabana , B. Holleran , M. E. Beaulieu , et al., “Critical Hydrogen Bond Formation for Activation of the Angiotensin II Type 1 Receptor,” Journal of Biological Chemistry 288, no. 4 (2013): 2593–2604, 10.1074/jbc.M112.395939. 23223579 PMC3554926

[42] Y. Shan , M. P. Eastwood , X. Zhang , et al., “Oncogenic Mutations Counteract Intrinsic Disorder in the EGFR Kinase and Promote Receptor Dimerization,” Cell 149, no. 4 (2012): 860–870, 10.1016/j.cell.2012.02.063. 22579287

[43] Y. Shan , E. T. Kim , M. P. Eastwood , R. O. Dror , M. A. Seeliger , and D. E. Shaw , “How Does a Drug Molecule Find Its Target Binding Site?,” Journal of the American Chemical Society 133, no. 24 (2011): 9181–9183, 10.1021/ja202726y. 21545110 PMC3221467

[44] J. M. Krieger , P. Doruker , A. L. Scott , D. Perahia , and I. Bahar , “Towards Gaining Sight of Multiscale Events: Utilizing Network Models and Normal Modes in Hybrid Methods,” Current Opinion in Structural Biology 64 (2020): 34–41, 10.1016/j.sbi.2020.05.013. 32622329 PMC7666066

[45] Q. Cui and I. Bahar , Normal Mode Analysis: Theory and Applications to Biological and Chemical Systems (CRC press, 2005).

[46] I. Bahar and A. Rader , “Coarse‐Grained Normal Mode Analysis in Structural Biology,” Current Opinion in Structural Biology 15, no. 5 (2005): 586–592. 16143512 10.1016/j.sbi.2005.08.007 PMC1482533

[47] D. A. Case , “Normal Mode Analysis of Protein Dynamics,” Current Opinion in Structural Biology 4, no. 2 (1994): 285–290.

[48] P. Doruker , A. R. Atilgan , and I. Bahar , “Dynamics of Proteins Predicted by Molecular Dynamics Simulations and Analytical Approaches: Application to Alpha‐Amylase Inhibitor,” Proteins 40, no. 3 (2000): 512–524. 10861943

[49] M. Gur , E. Zomot , and I. Bahar , “Global Motions Exhibited by Proteins in Micro‐ to Milliseconds Simulations Concur With Anisotropic Network Model Predictions,” Journal of Chemical Physics 139, no. 12 (2013): 121912, 10.1063/1.4816375. 24089724 PMC3739829

[50] I. Bahar , M. H. Cheng , J. Y. Lee , C. Kaya , and S. Zhang , “Structure‐Encoded Global Motions and Their Role in Mediating Protein‐Substrate Interactions,” Biophysical Journal 109, no. 6 (2015): 1101–1109, 10.1016/j.bpj.2015.06.004. 26143655 PMC4576147

[51] S. Subhadarshini , H. Tandon , N. Srinivasan , and R. Sowdhamini , “Normal Mode Analysis Elicits Conformational Shifts in Proteins at Both Proximal and Distal Regions to the Phosphosite Stemming From Single‐Site Phosphorylation,” ACS Omega 9, no. 23 (2024): 24520–24537, 10.1021/acsomega.4c00523. 38882086 PMC11170700

[52] T. Yamato and O. Laprevote , “Normal Mode Analysis and Beyond,” Biophysics and Physicobiology 16 (2019): 322–327, 10.2142/biophysico.16.0_322. 31984187 PMC6976091

[53] S. Maguid , S. Fernandez‐Alberti , L. Ferrelli , and J. Echave , “Exploring the Common Dynamics of Homologous Proteins. Application to the Globin Family,” Biophysical Journal 89, no. 1 (2005): 3–13, 10.1529/biophysj.104.053041. 15749782 PMC1366528

[54] C. Micheletti , “Comparing Proteins by Their Internal Dynamics: Exploring Structure–Function Relationships Beyond Static Structural Alignments,” Physics of Life Reviews 10, no. 1 (2013): 1–26. 23199577 10.1016/j.plrev.2012.10.009

[55] C. Chennubhotla , A. Rader , L.‐W. Yang , and I. Bahar , “Elastic Network Models for Understanding Biomolecular Machinery: From Enzymes to Supramolecular Assemblies,” Physical Biology 2, no. 4 (2005): S173. 16280623 10.1088/1478-3975/2/4/S12

[56] J. Echave , “Why Are the Low‐Energy Protein Normal Modes Evolutionarily Conserved?,” Pure and Applied Chemistry 84, no. 9 (2012): 1931–1937.

[57] S. Zhang , H. Li , J. M. Krieger , and I. Bahar , “Shared Signature Dynamics Tempered by Local Fluctuations Enables Fold Adaptability and Specificity,” Molecular Biology and Evolution 36, no. 9 (2019): 2053–2068, 10.1093/molbev/msz102. 31028708 PMC6736388

[58] M. L. Romero Romero , A. Rabin , and D. S. Tawfik , “Functional Proteins From Short Peptides: Dayhoff's Hypothesis Turns 50,” Angewandte Chemie International Edition 55, no. 52 (2016): 15966–15971. 27865046 10.1002/anie.201609977

[59] R. V. Eck and M. O. Dayhoff , “Evolution of the Structure of Ferredoxin Based on Living Relics of Primitive Amino Acid Sequences,” Science 152, no. 3720 (1966): 363–366. 17775169 10.1126/science.152.3720.363

[60] Z. Dauter , K. S. Wilson , L. C. Sieker , J. Meyer , and J. M. Moulis , “Atomic Resolution (0.94 A) Structure of Clostridium Acidurici Ferredoxin. Detailed Geometry of [4Fe‐4S] Clusters in a Protein,” Biochemistry 36, no. 51 (1997): 16065–16073, 10.1021/bi972155y. 9405040

[61] R. G. Parra , R. Espada , I. E. Sanchez , M. J. Sippl , and D. U. Ferreiro , “Detecting Repetitions and Periodicities in Proteins by Tiling the Structural Space,” Journal of Physical Chemistry B 117, no. 42 (2013): 12887–12897, 10.1021/jp402105j. 23758291 PMC3807821

[62] Y. Bromberg , A. A. Aptekmann , Y. Mahlich , et al., “Quantifying Structural Relationships of Metal‐Binding Sites Suggests Origins of Biological Electron Transfer,” Science Advances 8, no. 2 (2022): eabj3984, 10.1126/sciadv.abj3984. 35030025 PMC8759750

[63] A. Bakan , L. M. Meireles , and I. Bahar , “ProDy: Protein Dynamics Inferred From Theory and Experiments,” Bioinformatics 27, no. 11 (2011): 1575–1577, 10.1093/bioinformatics/btr168. 21471012 PMC3102222

[64] E. Eyal , L. W. Yang , and I. Bahar , “Anisotropic Network Model: Systematic Evaluation and a New Web Interface,” Bioinformatics 22, no. 21 (2006): 2619–2627, 10.1093/bioinformatics/btl448. 16928735

[65] P. J. Cock , T. Antao , J. T. Chang , et al., “Biopython: Freely Available Python Tools for Computational Molecular Biology and Bioinformatics,” Bioinformatics 25, no. 11 (2009): 1422–1423, 10.1093/bioinformatics/btp163. 19304878 PMC2682512

[66] J. C. Gower , “A General Coefficient of Similarity and Some of Its Properties,” Biometrics 27, no. 4 (1971): 857–871.

[67] J. Wilson , V. Borovitskiy , A. Terenin , P. Mostowsky , and M. Deisenroth , “Efficiently Sampling Functions From Gaussian Process Posteriors,” in International Conference on Machine Learning (PMLR, 2020), 10292–10302.

[68] L. L. Tan , R. H. Holm , and S. C. Lee , “Structural Analysis of Cubane‐Type Iron Clusters,” Polyhedron 58 (2013): 206–217, 10.1016/j.poly.2013.02.031. 24072952 PMC3780430

[69] N. W. Moriarty and P. D. Adams , “Iron–Sulfur Clusters Have no Right Angles,” Acta Crystallographica Section D: Structural Biology 75, no. 1 (2019): 16–20. 30644841 10.1107/S205979831801519X PMC6333285

[70] T. Wagner , U. Ermler , and S. Shima , “The Methanogenic CO_2_ Reducing‐and‐Fixing Enzyme Is Bifunctional and Contains 46 [4Fe‐4S] Clusters,” Science 354, no. 6308 (2016): 114–117. 27846502 10.1126/science.aaf9284

[71] D. Dobritzsch , S. Ricagno , G. Schneider , K. D. Schnackerz , and Y. Lindqvist , “Crystal Structure of the Productive Ternary Complex of Dihydropyrimidine Dehydrogenase With NADPH and 5‐Iodouracil: IMPLICATIONS FOR MECHANISM OF INHIBITION AND ELECTRON TRANSFER,” Journal of Biological Chemistry 277, no. 15 (2002): 13155–13166. 11796730 10.1074/jbc.M111877200

[72] T. Wagner , J. Koch , U. Ermler , and S. Shima , “Methanogenic Heterodisulfide Reductase (HdrABC‐MvhAGD) Uses Two Noncubane [4Fe‐4S] Clusters for Reduction,” Science 357, no. 6352 (2017): 699–703. 28818947 10.1126/science.aan0425

[73] S. F. Altschul , W. Gish , W. Miller , E. W. Myers , and D. J. Lipman , “Basic Local Alignment Search Tool,” Journal of Molecular Biology 215, no. 3 (1990): 403–410, 10.1016/S0022-2836(05)80360-2. 2231712

[74] W. R. Pearson , “An Introduction to Sequence Similarity (“Homology”) Searching,” Current Protocols in Bioinformatics Chapter 3 (2013): 3.1.1–3.1.8, 10.1002/0471250953.bi0301s42. PMC3820096 23749753

[75] E. G. Emberly , R. Mukhopadhyay , C. Tang , and N. S. Wingreen , “Flexibility of β‐Sheets: Principal Component Analysis of Database Protein Structures,” Proteins: Structure, Function, and Bioinformatics 55, no. 1 (2004): 91–98. 10.1002/prot.10618 14997543

[76] E. G. Emberly , R. Mukhopadhyay , N. S. Wingreen , and C. Tang , “Flexibility of α‐Helices: Results of a Statistical Analysis of Database Protein Structures,” Journal of Molecular Biology 327, no. 1 (2003): 229–237. 12614621 10.1016/s0022-2836(03)00097-4

[77] A. Hochheimer , R. A. Schmitz , R. K. Thauer , and R. Hedderich , “The Tungsten Formylmethanofuran Dehydrogenase From Methanobacterium Thermoautotrophicum Contains Sequence Motifs Characteristic for Enzymes Containing Molybdopterin Dinucleotide,” European Journal of Biochemistry 234, no. 3 (1995): 910–920. 8575452 10.1111/j.1432-1033.1995.910_a.x

[78] R. Hedderich , S. Albracht , D. Linder , J. Koch , and R. Thauer , “Isolation and Characterization of Polyferredoxin From Methanobacterium Thermoautotrophicum the Mvhb Gene Product of the Methylviologen‐Reducing Hydrogenase Operon,” FEBS Letters 298, no. 1 (1992): 65–68. 1312016 10.1016/0014-5793(92)80023-a

[79] A. Hochheimer , D. Linder , R. K. Thauer , and R. Hedderich , “The Molybdenum Formylmethanofuran Dehydrogenase Operon and the Tungsten Formylmethanofuran Dehydrogenase Operon From Methanobacterium Thermoautotrophicum: Structures and Transcriptional Regulation,” European Journal of Biochemistry 242, no. 1 (1996): 156–162. 8954165 10.1111/j.1432-1033.1996.0156r.x

[80] S. S. Krishna , R. I. Sadreyev , and N. V. Grishin , “A Tale of Two Ferredoxins: Sequence Similarity and Structural Differences,” BMC Structural Biology 6 (2006): 8, 10.1186/1472-6807-6-8. 16603087 PMC1459171

[81] R. K. Thauer , A. K. Kaster , H. Seedorf , W. Buckel , and R. Hedderich , “Methanogenic Archaea: Ecologically Relevant Differences in Energy Conservation,” Nature Reviews. Microbiology 6, no. 8 (2008): 579–591, 10.1038/nrmicro1931. 18587410

[82] A. K. Kaster , J. Moll , K. Parey , and R. K. Thauer , “Coupling of Ferredoxin and Heterodisulfide Reduction via Electron Bifurcation in Hydrogenotrophic Methanogenic Archaea,” Proceedings of the National Academy of Sciences of the United States of America 108, no. 7 (2011): 2981–2986, 10.1073/pnas.1016761108. 21262829 PMC3041090

[83] D. C. Rathod , S. M. Vaidya , M. T. Hopp , T. Kuhl , and D. Imhof , “Shapes and Patterns of Heme‐Binding Motifs in Mammalian Heme‐Binding Proteins,” Biomolecules 13, no. 7 (2023): 1–13, 10.3390/biom13071031. PMC10377097 37509066

[84] T. Li , H. L. Bonkovsky , and J. T. Guo , “Structural Analysis of Heme Proteins: Implications for Design and Prediction,” BMC Structural Biology 11 (2011): 13, 10.1186/1472-6807-11-13. 21371326 PMC3059290

[85] W. Zheng , “Anharmonic Normal Mode Analysis of Elastic Network Model Improves the Modeling of Atomic Fluctuations in Protein Crystal Structures,” Biophysical Journal 98, no. 12 (2010): 3025–3034, 10.1016/j.bpj.2010.03.027. 20550915 PMC2884254

