# Supplementary material for: Comparative Dynamics Enables Discovery of Embedded Bacterial Ferredoxin Domains in Large Redox Enzymes
Source: Proteins. 2025 Jun 19;93(11):1973–87. doi: 10.1002/prot.70004 (PMC12517254; doi:10.1002/prot.70004)
Supplement: Supplementary file 1 — Data S1. Supporting Information. [file PROT-93-1973-s001.docx]

**Supporting Information for**

Comparative Dynamics Enables Discovery of Embedded Bacterial Ferredoxin Domains in Large Redox Enzymes

**Short Running Title:** Dynamics of Embedded Ferredoxin Domains

Jan A. Siess, Vikas Nanda*

Department of Biochemistry and Molecular Biology, Robert Wood Johnson Medical School and the Center for Advanced Biotechnology and Medicine, Rutgers, The State University of New Jersey, Piscataway, NJ*Vikas Nanda

**Email:**  [*vik.nanda@rutgers.edu](mailto:*vik.nanda@rutgers.edu), [jsiess93@gmail.com](mailto:jsiess93@gmail.com)

**This PDF file includes:**

Figures S1 to S6

Table S1

**Supplementary Figures**


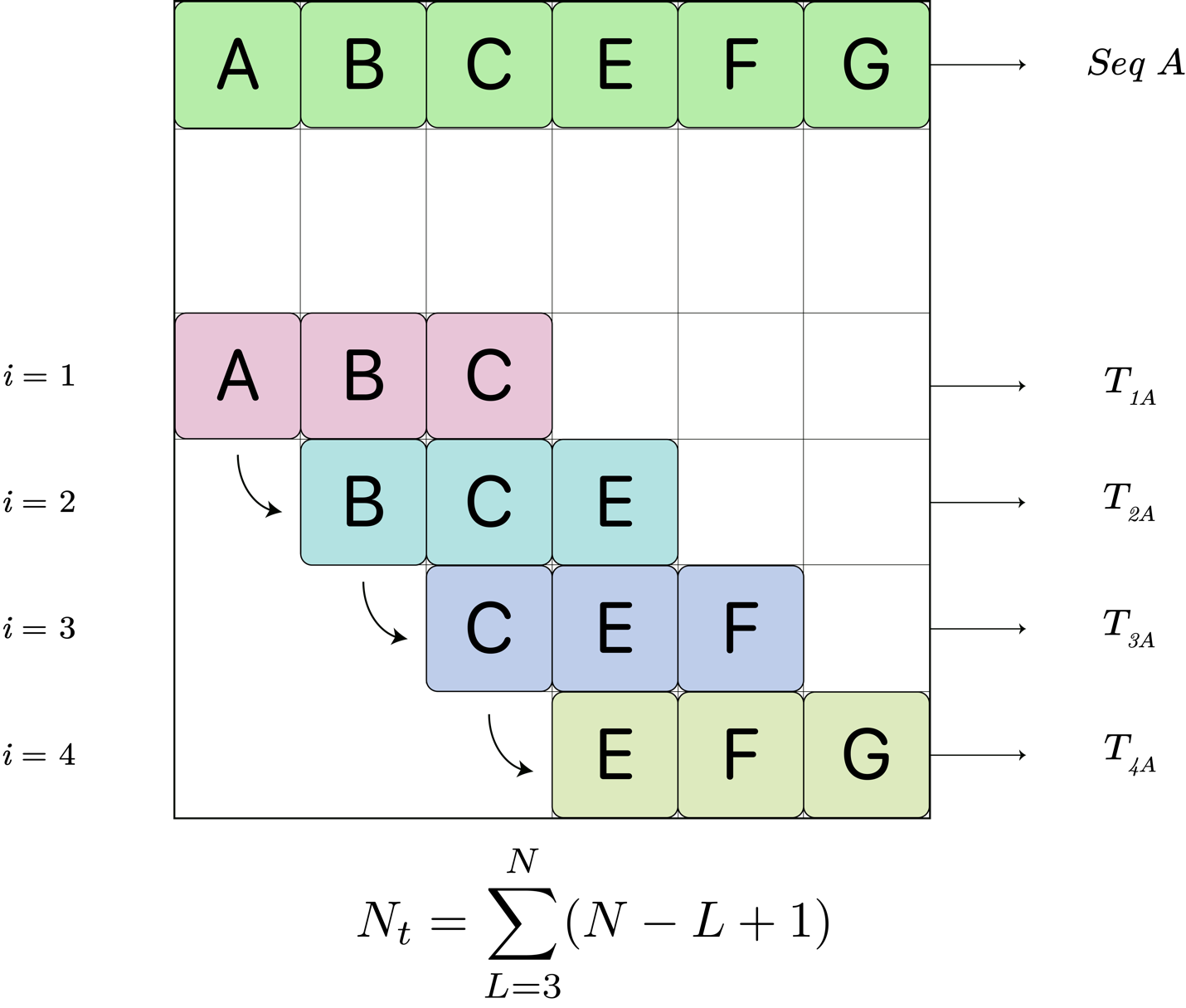


**Fig. S1: Creating Tiles Using a Sliding Window Approach.** This figure illustrates the process of generating tiles using a sliding window approach. Starting with a minimum tile length *L*, we iteratively create tiles T_i_ for each position *i*, until the maximum protein length *N* is reached. The example depicted above demonstrates working with a sequence of length 6 and generating tiles beginning from a length of 3, resulting in a total of 4 tiles. Once those tiles have spanned the entire sequence, *L* is incremented by 1, and the process repeats in the same fashion until *L* equals *N*.

**Fig. S2: Extraction of query (Q) and target (T) sub-matrices and evaluation of the Frobenius distance.** (**A**) Independent ANM calculations yield an *N* *x* *N* cross-correlation matrix for a hypothetical protein A (**C_A_**) and protein B (**C_B_**). (**B**) A contiguous tile of length L = 3 (residues k…k + L – 1) is selected from each matrix, producing two *L x L* sub-matrices: **Q** = **C_A_**[k:k + L, k:k + L] and **T** = **C_B_**[k:k + L, k:k + L]. (**C**) The difference matrix **D** is formed and its Frobenius norm is calculated, providing a scalar measure of the dynamical dissimilarity between two tiles.


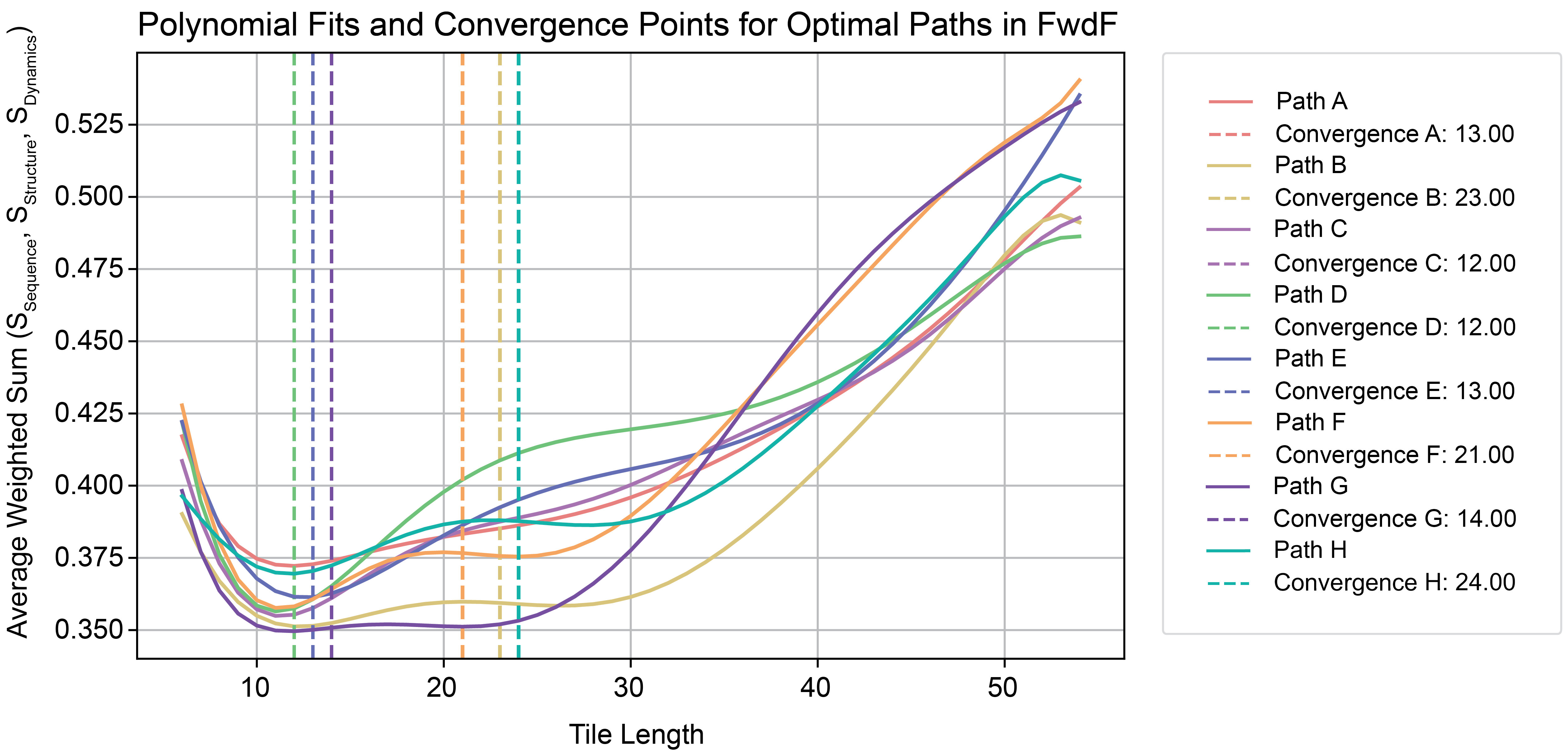


**Fig. S3: Polynomial Fits and Tile Length Convergence Points for Optimal Paths in FwdF.** This figure illustrates the polynomial fits and corresponding convergence points for the optimal paths identified in FwdF. Each colored line represents the polynomial fit of the average weighted sum as a function of tile length for a specific path, with the polynomial degree determined by minimizing the Bayesian Information Criterion (BIC). The vertical dashed lines indicate the convergence points for each path, where the derivative of the polynomial stabilizes, identified by detecting the minimum rate of change in the smoothed derivative. These convergence points, determined using a moving average and Gaussian smoothing, represent the tile lengths where the balance between sufficient sequence coverage and presumed biological significance is most effectively achieved.


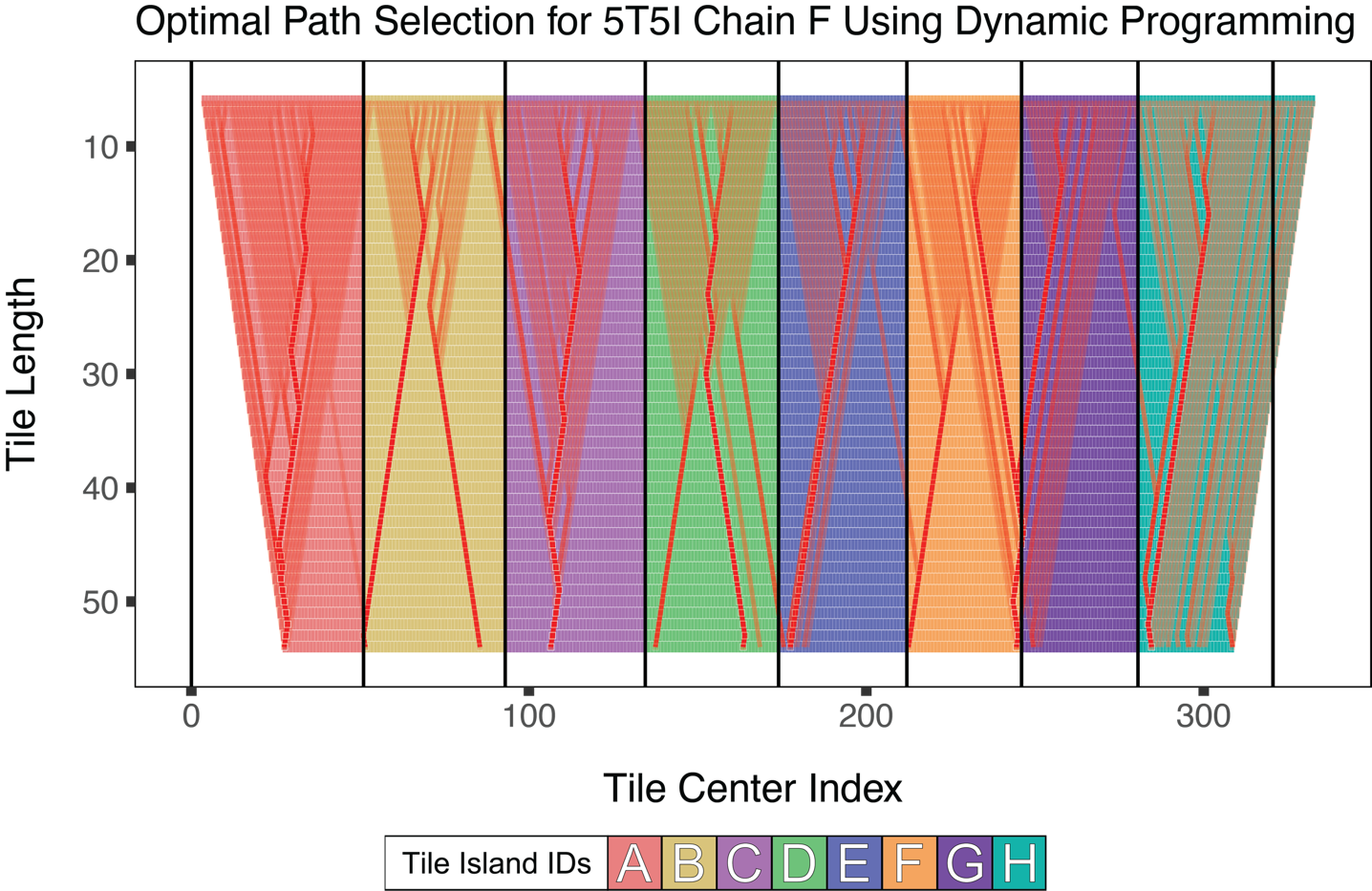


**Fig. S4: Control Experiment Featuring FwdF**. Chain F of Fwd was used as a positive control due to presence of distinct ferredoxin domains. In this figure, the cysteine bonding constraint has been removed, leading to two key observations. First, without the cysteine bonding constraint, the paths naturally organize into their respective islands. Second, the optimal paths for each island, despite some bifurcations and minor differences, are congruent with those observed in the positive control.


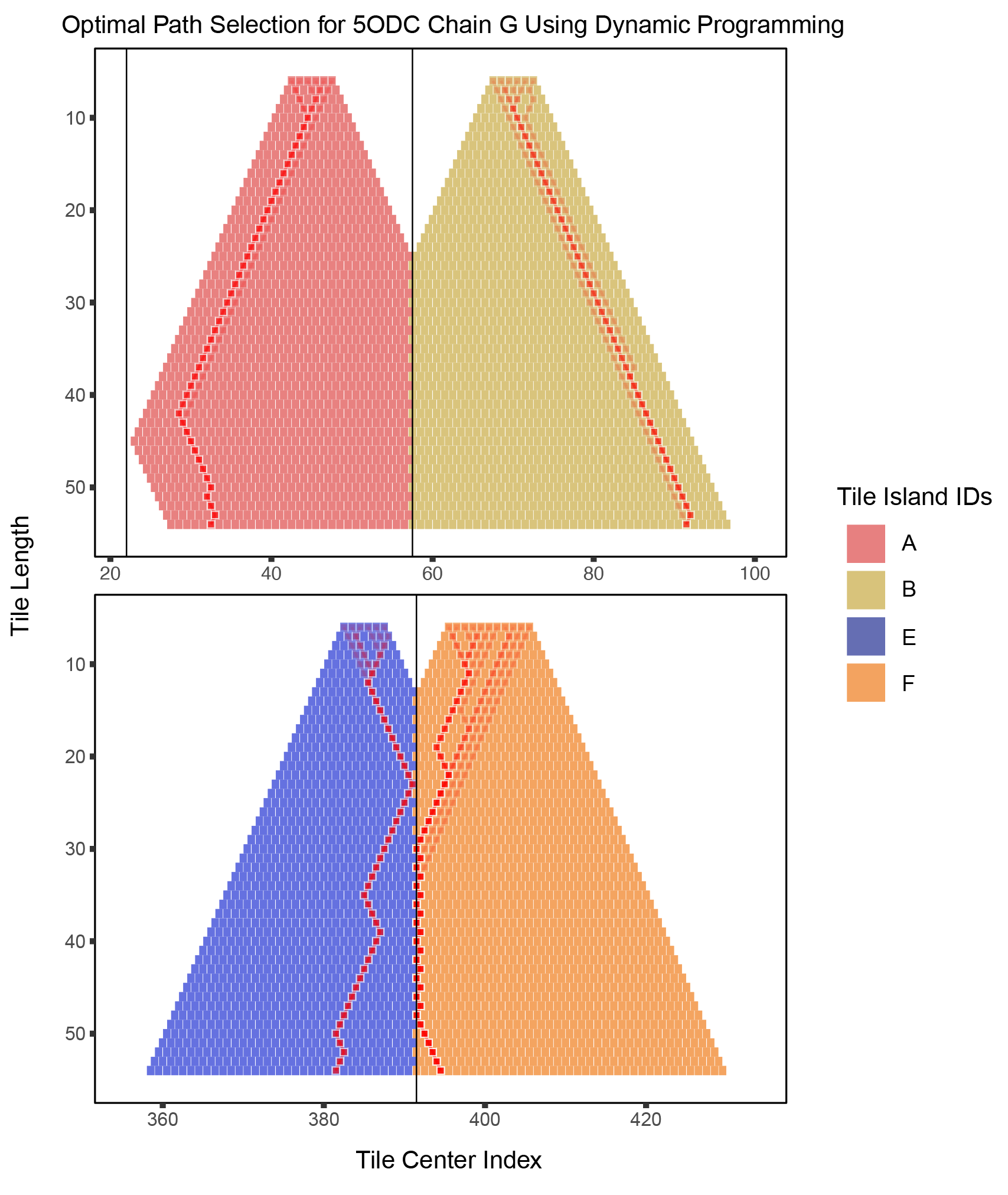


**Fig. S5: Optimal paths for islands A, B, E, and F from Chain G of Hdr.** This figure depicts the optimal paths, identified by the dynamic programming algorithm, for islands A, B, E, and F in chain G of Hdr. The optimal path is illustrated above with red lines and squares. The combinations of tile length and center indices along this path map onto portions of chain G which resemble ferredoxins inserted within Rossmann folds.

**Fig. S6: Concave baseline is metric-imposed.** The upper panel shows every observed tile-pair from chain F of 5T5I, plotted as the cosine similarity of their ANM cross-correlation vectors against the same pairs Frobenius distances; the red curve is generated via cubic smoothing splines. The lower panel is a control in which we keep only the metric geometry: two tile norms *r_Q_*, *r_T_* are resampled from the empirical norm distribution, an independent cosine value $\rho$ is drawn, and the analytic law of cosines $d_{Frobenius}=\sqrt{r_{Q}^{2}+r_{T}^{2}-2r_{Q}r_{T}\rho}$ is applied. The synthetic cloud reproduces the characteristic √-shaped curvature, confirming that a non-linear relationship is mandated by combining a bounded angular similarity with an RMS-type distance; the much sharper slope in the real data must arise from genuine structure-dynamics correlations, not from artifacts of the chosen metrics.

**Table S1: Residue Ranges for Optimal Paths Across Target Structures’ Tile Islands.** This table details the residue ranges defining the optimal paths through each tile island in the target structures analyzed. The residue ranges are based on half the length of the query protein, which constrains the algorithm's search space and increases the likelihood of identifying biologically significant matches rather than coincidental ones. While this length heuristic is used to guide the search, methods such as those outlined in the first supplement provide a more robust, autonomous approach to optimizing tile length.
